# Unveiling genetic insights: Array-CGH and WES discoveries in a cohort of 122 children with essential autism spectrum disorder

**DOI:** 10.1186/s12864-024-11077-5

**Published:** 2024-12-10

**Authors:** Paola Granata, Alessandra Zito, Dario Cocciadiferro, Antonio Novelli, Chiara Pessina, Tommaso Mazza, Matteo Ferri, Paolo Piccinelli, Chiara Luoni, Cristiano Termine, Mauro Fasano, Rosario Casalone

**Affiliations:** 1 Cytogenetics and Medical Genetics Unit, Department of Services, ASST dei Sette Laghi, Varese, Italy; 2 School of Medical Genetics, Department of Medicine and Surgery, University of Insubria, Varese, Italy; 3 Translational Cytogenomics Research Unit, Ospedale Pediatrico Bambino Gesù, Roma, Italy; 4 Laboratory of Bioinformatics, IRCCS Casa Sollievo della Sofferenza, S. Giovanni, Rotondo, Italy; 5 Child Neuropsychiatry Unit, Department of Maternal and Child Health, ASST dei Sette Laghi, Varese, Italy; 6 Child Neuropsychiatry Unit, Department of Medicine and Surgery, University of Insubria, Varese, Italy; 7 Department of Science and High Technology, University of Insubria, via Manara 7, Busto Arsizio, Italy; 8 Center of Neuroscience, University of Insubria, Busto Arsizio, Italy

**Keywords:** Essential autistic spectrum disorder, ASD, Array-comparative genomic hybridization (array-CGH), Copy number variants (CNVs)

## Abstract

**Background:**

Autistic Spectrum Disorder (ASD) is a neurodevelopmental disorder with a strong genetic component and high heterogeneity. Essential ASD refers to patients who do not have other comorbidities. This study aimed to investigate the genetic basis of essential ASD using whole exome sequencing (WES) and array-comparative genomic hybridization (array-CGH).

**Results:**

In a cohort of 122 children with essential ASD, WES detected 382 variants across 223 genes, while array-CGH identified 46 copy number variants (CNVs). The combined use of WES and array-CGH revealed pathogenic variants in four patients (3.1% detection rate) and likely pathogenic variants in 34 patients (27.8% detection rate). Only one patient had a pathogenic CNV (0.8% detection rate). Including likely pathogenic variants, the overall detection rate was 31.2%. Additionally, 33 *de novo* heterozygous sequence variants were identified by WES, with three classified as pathogenic and 13 as likely pathogenic. Sequence variants were found in 85 genes already associated with ASD, and 138 genes not previously included in the SFARI dataset were identified as potential new candidate genes.

**Conclusions:**

The study enhances genetic understanding of essential ASD and identifies new candidate genes of interest. The findings suggest that using both array-CGH and WES in patients with essential ASD can improve the detection of pathogenic and likely pathogenic genetic variants, contributing to better diagnosis and potentially guiding future research and treatment strategies.

**Supplementary Information:**

The online version contains supplementary material available at 10.1186/s12864-024-11077-5.

## Background

Autistic Spectrum Disorder (ASD) includes a diverse group of neurodevelopmental disorders characterized by deficits in social communication and interaction, along with restricted and repetitive behaviors, interests, and activities. The prevalence of ASD in the general population is approximately 1%. Many patients with ASD also exhibit global developmental delay (GDD) and epilepsy, leading to the classification of “complex ASD”. Other patients may have specific patterns of abnormalities or dysmorphic features, referred to as “syndromic ASD”, which includes conditions like Fragile X syndrome, Rett syndrome, Down’s Syndrome, Phenylketonuria, Angelman syndrome, and Tuberous Sclerosis Complex [1–3]. Non-syndromic ASD refers to patients without dysmorphic traits, comorbidities, or a characteristic symptom pattern of a specific syndrome. ASD can be categorized into three subtypes: essential ASD (three or fewer anomalies), equivocal ASD (four or five anomalies), and complex ASD (six or more anomalies) [3]. Males are more frequently affected than females, with a male-to-female ratio of 2.5:1 for complex ASD [4] and 4.5:1 for essential ASD [5].

Array-comparative genomic hybridization (array-CGH) has been integrated into genetic diagnostics for ASD, identifying copy number variations (CNVs) that contribute to approximately 10% of ASD cases [6]. Variants of uncertain significance (VOUS) are also frequently identified, with *de novo* CNVs present in about 4% of ASD patients [7]. CNVs related to neuronal cell adhesion, ubiquitin pathways [8], and postsynaptic cell adhesion [9] are biologically associated with ASD. The Simons Foundation Autism Research Initiative (SFARI) database lists recurrent CNVs associated with ASD [10]. Previous studies on array-CGH in essential ASD have shown a causative CNV detection rate (DR) of 9%, with no significant difference in cognitive abilities among groups with and without causative CNVs [11]. However, a higher number of non-verbal children were observed in the causative CNV group. High-resolution array-CGH findings in essential ASD have reported lower frequencies of pathogenic CNVs compared to complex ASD, with no correlation between genetic results and clinical aspects. In high-functioning autism (HFA) without epilepsy, intellectual disability, or known genetic diseases, array-CGH identified CNVs containing brain-related genes but found no difference in the number of CNVs compared to random population samples [12]. In non-syndromic autism with epilepsy, intellectual disability, and ADHD, array-CGH identified *de novo* pathogenic CNVs in 6.25% of patients [13] .

Next-generation sequencing techniques, such as whole exome sequencing (WES) and whole genome sequencing (WGS), have identified hundreds of gene variants involved in ASD, highlighting the role of *de novo* sequence variants [14, 15]. A combination of WES and array-CGH in essential ASD has identified clinically significant variants in 5.9% of cases, with pathogenic CNVs found in 4.2% and WES-positive findings in 3.1% of essential ASD patients [13, 16]. Interestingly, while hundreds of ASD loci have been identified, the genetic basis of ASD remains elusive, and only a small fraction of ASD patients have been associated with specific genetic variants. Other factors, such as rare genetic variations, epigenetic changes, gene-gene interactions, or environmental factors, may also play a role in the development of ASD, highlighting the need for continued research to better understand the genetic and non-genetic factors that contribute to the disease.

In this study, we analyzed 122 children with essential ASD and their parents using array-CGH and WES. We report the molecular findings from this dual testing strategy and discuss the potential pathogenicity and clinical significance of the results.

## Methods

### Participants

We enrolled 122 patients (104 males and 18 females) with a diagnosis of essential ASD. These patients had no epilepsy, dysmorphic features, intellectual disability, microcephaly, six or more minor anomalies, or systemic congenital malformations such as congenital heart defects. Patients were recruited from the Child Neuropsychiatry Unit and the Cytogenetics and Medical Genetics Unit of “ASST Sette Laghi”, Varese, Italy. A clinical geneticist assessed heritability for neuropsychiatric disorders and neurodevelopmental diseases and excluded multiple congenital abnormalities, malformations, or syndromes. Subjects aged from three to 12 years, with an ASD diagnosis based on DSM-5 criteria, were included. Exclusion criteria were: (a) demonstrated syndromic ASD with a genetic basis or up to six other clinical features (complex ASD); (b) absence of one or both parents; (c) known syndromes related to specific genetic causes; (d) presence of epilepsy or use of epileptic pharmacological therapy, or febrile seizures within six months prior to medical counseling; (e) other psychopharmacological therapies. Written informed consent for genetic and clinical ASD tests and the use of biological results for research in an anonymous form was provided by the parents and relatives of the probands. The consent model and procedure were approved by the Institutional Review Board (“ASST Sette Laghi” Code MOD09 IOS01SSDGM).

### Genetic investigation

DNA from peripheral blood cells has been selected as elective tissue for the genetic investigations. As a matter of fact, even if limited tissue mosaicism has been associated to syndromic neurodevelopmental disorders, it has not been confirmed in ASD affected patients [17, 18].

To identify submicroscopic chromosomal rearrangements, array-CGH technology was performed after DNA extraction from peripheral blood cells (QIAmp DNA Blood Maxi Kit, Qiagen, Hilden, Germany). The array-based comparative genomic hybridization was performed using the CytoSure ISCA V3 4 × 180 K platform, with a backbone resolution of 1 probe/22 Kb for high-priority backbone, 1 probe/24 Kb for medium-priority backbone, and 1 probe/54 Kb for low-priority backbone, using the human genome reference GRCh37/hg19 and sex-matched normal human DNA pool (Kreatech, Amsterdam, Holland) as control. The InnoScan 710 Microarray Scanner (Carbonne, France) and Mapix (Innopsys, Carbonne, France) were used to detect and analyze fluorescence levels. Results were interpreted using Cytosure Interpret Software (Oxford Gene Technology, Begbroke, Oxfordshire, United Kingdom). QC metrics required were SD < 1.0 and DLR spread < 0.3.

### Whole exome sequencing

WES was performed in trios using the Twist Human Core Exome Kit (Twist Bioscience, San Francisco, USA) according to the manufacturer’s protocol and sequenced with the Illumina NovaSeq 6000 platform. The BaseSpace pipeline (Illumina, San Diego, USA) and TGex software (LifeMap Sciences, Alameda, USA) were used for variant calling and annotation, respectively. Sequencing data were aligned to the GRCh37/hg19 human reference genome. Variants with a coverage lower than 10×, quality score (GQ) lower than 15, and gnomAD minor allele frequency (MAF) lower than 5% were excluded. WES results were interpreted according to ACMG guidelines [19]. The prediction of the effect of a single base variant on protein structure and functionality was determined by the CADD score, using genome build GRCh37/hg19 v1.4 as reference. Annotations were performed using gnomAD, GeneCards, UniProtKB/Swiss-Prot, OMIM, GTEx, SFARI, CADD, and HGNC databases.

### Gene filtering

WES was used to investigate 825 neurodevelopmental genes from the SFARI database (2018 Q3 version). Additionally, all other genes, whether included in the OMIM database or not, and not reported in the SFARI database, were considered if the analytic software detected *de novo*, compound heterozygous, truncating, frameshift, or splicing variants with MAF less than 1% in the general population (gnomAD database v2.1.1 and v3.1).

### Sequence variant filtering

All synonymous variants and inherited heterozygous variants were excluded. Hemizygous, homozygous, compound heterozygous, and *de novo* variants with ≤ 10 homozygotes in the general population or not annotated in the gnomAD database (v2.1.1 and v3.1) were considered. Additionally, compound heterozygous variants were included if one variant was classified as likely pathogenic (LP) and the second variant was classified as variant of uncertain significance (VOUS), LP, or pathogenic (P), regardless of the number of homozygotes in population databases.

### Pathogenic evaluation criteria of sequence variants

Variants were classified as likely benign (LB), VOUS, LP, or P according to the following criteria:

Variants in SFARI genes were classified as P if they had a CADD score ≥ 20 (criterion 1), an ACMG evaluation of P or LP (criterion 2), and were not found in the gnomAD database or had zero or one homozygote in the general population (criterion 3).

A variant was considered LP if it had two to ten homozygous subjects in the general population and satisfied criterion 1, with ACMG evaluation of P, LP, or VOUS, and involvement in neurodevelopment, nervous system function, synaptic transmission, or epigenetic transcription regulation (criterion 4).

Variants with a CADD score < 15 and ACMG evaluation of LB were classified as LB or B.

Variants not meeting these criteria were classified as VOUS.

For genes not in the SFARI database, variants were classified as LP if they met criteria 1, 2, and 4. They were classified as LB or B with ACMG evaluation of B, LB, or VOUS and a CADD score < 15. Variants in non-SFARI genes were conservatively not classified as P due to their unknown implication in the ASD phenotype.

Compound heterozygous conditions were defined by considering the combination of the pathogenicity classification of single variants as shown in Table 1.

**Table 1:** Classification of compound heterozygosity conditions

Variant 1	Variant 2	Classification
P	P or LP	P
LP	LP or VOUS	LP
VOUS	LB or VOUS	VOUS
LB	LP or P	VOUS
LB	LB	LB

Considering that most variants in neurodevelopmental disorders are susceptibility factors with incomplete penetrance [20, 21], we considered incomplete penetrance for variants found in all patients to determine their pathogenicity.

### Copy number variants (CNVs) interpretation and classification

CNVs were interpreted using public databases like DECIPHER (including dosage sensitivity scores and sampling probability) and the Database for Genetic Variants (DGV). Classification followed the American College of Medical Genetics (ACMG) Joint Consensus [22] and Cytogenetic European and International Guidelines [23], categorizing CNVs as P, LP, VOUS, LB, or B. CNVs classified as B or LB and those present in more than 1% of healthy subjects in the DGV database were excluded.

### Criteria for “Genes of interest” definition in CNVs

Genes involved in monogenic CNVs were considered of interest. In multiple-gene CNVs, candidate genes were selected based on SFARI score, function, involvement in neurodevelopment, brain expression levels (TGEx), and dosage sensitivity.

### Data processing

All WES and array-CGH data were analyzed considering variant effects, pathogenicity classification, zygosity, recurrence, and gene families identified via GeneCards [24]. Non-annotated variants in the gnomAD database and genes not reported in SFARI were emphasized. Detection rates for the two combined tests in the 122 patients with Essential Autism were estimated based on P and LP variants.

### Selection of genes for gene families

Gene families with more than five genes were analyzed. Genes that were recurrent and not annotated in the SFARI database were considered. Family identification and selection were performed using information from GeneCards (genecards.com) and UniProt (uniprot.com).

## Results

Genetic analysis of DNA samples extracted from blood tissue of 122 ASD subjects revealed a positive result for a genetic variant based on variant calling exclusively from WES in 74 samples, while 30 showed positive results from both WES and array-CGH.

Five individuals tested positive only on array-CGH, and 13 had negative results on genetic testing. The cohort consisted of 104 males and 18 females, resulting in a male-to-female ratio of 5.8:1. Descriptive statistics and detection rates are reported in Table 2, compared with previous studies.

**Table 2:** Detection rates of sequence and copy number variants

Category	This study	Other Studies
**Pathogenic variants**	3.3% (4 cases)	5.9% (25) 6.3% (16)
- Pathogenic sequence variants	2.5% (3 cases)	3.1% (16)
- Pathogenic CNVs	0.82% (1 case)	3.1% (11), 9% (2), 4.2% (16)
**Likely Pathogenic (LP) variants**	27.9% (34 cases)	
- LP sequence variants	19.7% (24 cases)	
- LP CNVs	6.5% (8 cases)	
- LP sequence variants & CNVs	1.6% (2 cases)	
**Variants of Uncertain Significance (VOUS)**	57.4% (70 cases)	
- VOUS sequence variants	44.3% (54 cases)	
- VOUS CNVs	2.5% (3 cases)	
- VOUS sequence variants & CNVs	10.6% (13 cases)	
**No variants or benign/likely benign**	11.5% (14 cases)	
**Overall Detection Rate with LP variants**	30.8% (32 cases)	

A total of 381 sequence variants across 223 genes were identified in 104 patients using Whole Exome Sequencing (WES), including 317 non-synonymous missense variants, 16 frameshift, seven indels, 32 splicing variants, four start codon changes, and five stop codon changes (see Supplementary Table 1). Table 3 reports a summary of WES data.

**Table 3:** Summary of sequence variants identified by WES

Category	Number of Variants
**Total Variants Identified (WES)**	381
- Non-synonymous Missense	317
- Frameshift	16
- Indels	7
- Splicing Variants	32
- Start Codon Changes	4
- Stop Codon Changes	5
**Classification of Variants**	
- Pathogenic	4
- Likely Pathogenic (LP)	34
- Variants of Uncertain Significance (VOUS)	289
- Likely Benign (LB)	54
***de novo*** **Heterozygous Variants**	**33 variants (28 cases)**
- Pathogenic	3
- Likely Pathogenic (LP)	13
- Variants of Uncertain Significance (VOUS)	17
**Hemizygous Variants**	**91 variants (56 cases)**
- Likely Pathogenic (LP)	10
- Variants of Uncertain Significance (VOUS)	66
- Likely Benign (LB)	15
**Homozygous Variants**	**14 variants (10 cases)**
- Likely Pathogenic (LP)	2
- Variants of Uncertain Significance (VOUS)	12
- Likely Benign (LB)	1
**Compound Heterozygous Variants**	**240 variants (70 cases)**
- Pathogenic	1
- Likely Pathogenic (LP)	6
- Variants of Uncertain Significance (VOUS)	102
- Likely Benign (LB)	10
**Private Variants (Unique)**	**95 variants (61 patients)**
- Pathogenic	2 (2.1%)
- Likely Pathogenic (LP)	24 (25.3%)
- Variants of Uncertain Significance (VOUS)	61 (64.2%)
- Likely Benign (LB)	8 (8.4%)

Moreover, 46 CNVs were detected in 36 patients (see Supplementary Table 2). A summary of these variants is shown in Table 4. Combining the 259 genes with selected variants detected across both genetic tests, 32 gene families were represented (see Table 5).

**Table 4:** Summary of copy number variants identified by aCGH

CNV Type	Total Variants (%)
Deletions	16 (34.8%)
Duplications	30 (65.2%)
Pathogenic/Likely Pathogenic	12 (9.8%)
VOUS	34 (73.9%)

**Table 5:** Gene families highlight. Gene families were selected from Uniprot/Swissprot and HGNC database

Patient	Gene name	Gene families	Annotatation in SFARI db	Number of genes
A070	*ABCA13*	ABC transporter superfamily	YES	4
A111	*ABCA13*		YES	
A115	*ABCB7*		NO	
A016	*ABCC1*		NO	
A016	*ABCC6*		NO	
A013	*SLC12A3*		NO	3
A060	*SLC25A6*		NO	
A008	*SLC35A2*		NO	
A026	*ACTL9*	actin family	NO	2
A076	*ACTR5*	actin family	NO	
A124	*ACAD10*	acyl-CoA dehydrogenase family	NO	2
A060	*ACAD8*		NO	
A116	*ANK3*	Ankyrin family	YES	2
A052	*ANK2*		YES	
A005	*FAT1*	Cadherin related	YES	2
A044	*FAT1*		YES	
A033	*PCDH15*		YES	
A025	*CDH13*	cadherins Major	YES	1
A114	*CDH22*	cadherins Type II classical	YES	2
A017	*CDH8*		YES	
A010	*CACNA1F*	calcium channel alpha-1 subunit (TC 1.A.1.11) family	YES	3
A107	*CACNA1F*		YES	
A011	*CACNA1G*		YES	
A029	*CACNA2D1*	calcium channel subunit alpha-2/delta family	YES	1
A121	*SCN2A*	sodium channel (TC 1.A.1.10) family	YES	2
A046	*SCN9A*		YES	
A124	*MAPKAPK5*	protein kinase family CAMK Ser/Thr	NO	3
A084	*PASK*		YES	
A080	*PNCK*		NO	
A028	*MAP3K15*	protein kinase family STE Ser/Thr	NO	3
A099	*PAK2*		YES	
A130	*PAK3*		NO	
A084	*COL16A1*	Collagens family	NO	2
A027	*COL4A6*		NO	
A122	*SEC24B*	COPII coat complex	NO	2
A024	*SEC31B*		NO	
A079	*DDX20*	helicase family DEAD box	NO	3
A060	*DDX53*		YES	
A131	*DDX53*		YES	
A125	*DHX8*		NO	
A006	*DHX8*		NO	
A003	*ATRX*	helicase family SNF2/RAD54	YES	5
A050	*CHD1L*		NO	
A048	*CHD7*		YES	
A075	*EP400*		YES	
A034	*EP400*		YES	
A103	*EP400*		YES	
A109	*EP400*		YES	
A079	*SMARCA1*		NO	
A110	*GPR50*	G-protein coupled receptor 1 family	NO	4
A003	*HTR2A*		NO	
A026	*OR2Z1*		NO	
A060	*P2RY8*		NO	
A108	*CASR*	G-protein coupled receptor 3 family	NO	2
A118	*GABBR1*		NO	
A075	*KIF13B*	Kinesin family	YES	2
A074	*KIF15*		NO	
A051	*LRP1*	LDLR family	YES	2
A021	*LRP2*		YES	
A120	*LRP2*		YES	
A006	*MAGEC1*	MAGE family	NO	2
A057	*MAGEE2*		NO	
A058	*MAGEE2*		NO	
A059	*MAGEE2*		NO	
A060	*MAP7D2*	MAP7 family	NO	2
A026	*MAP7D3*		NO	
A011	*NRXN1*	neurexin family	YES	2
A120	*NRXN3*		YES	
A093	*PLXNA3*	plexin family	YES	2
A099	*PLXNA3*		YES	
A010	*PLXNB3*		NO	
A016	*MARF1*	Protein phosphatase 1 regulatory subunits	NO	3
A025	*PREX2*		NO	
A021	*YLPM1*		NO	
A002	*PPP2R1B*	Protein phosphatase 2 regulatory subunits	YES	2
A097	*PPP2R3B*		NO	
A014	*PTPRB*	protein-tyrosine phosphatase family	YES	2
Z008	*PTPRF*		NO	
A062	*RNF19A*	Ring finger proteins	NO	3
A097	*RNF220*		NO	
A100	*RNF25*		YES	
A065	*TRIM32*	TRIM/RBCC family	YES	2
A095	*TRIM35*		NO	
A088	*CRLF2*	cytokine receptor type I family-	NO	2
A119	*CRLF2*		NO	
A060	*IL3RA*		NO	
A095	*VCX*	VCX/VCY family	NO	2
A084	*VCX3B*		NO	
A111	*LLGL1*	WD repeat L(2)GL family	NO	2
A071	*STXBP5*		YES	
A014	*HIVEP2*	Zinc fingers C2H2-type family	YES	11
A044	*HIVEP3*		YES	
A008	*KLF8*		NO	
A029	*PRDM15*		NO	
A026	*ZHX1*		NO	
A020	*ZIC1*		NO	
A020	*ZIC4*		NO	
A125	*ZNF462*		YES	
A027	*ZNF630*		YES	
A051	*ZSCAN21*		NO	
A006	*ZMYM2*	Zinc fingers MYM-type family	NO	3
A006	*ZMYM5*		NO	
A113	*ZMYM5*		NO	
A093	*SMYD1*		NO	

## Discussion

### Detection rate

The overall detection rate (DR) of 3.3% for pathogenic variants in this cohort matches prior findings but is lower compared to some studies [2, 11, 16, 25]. The disparity in DR between different studies may be attributed to varying classification criteria and testing methodologies. When likely pathogenic variants (LP) are included, the detection rate increased substantially to 30.8%, reflecting the potential clinical relevance of LP variants in understanding ASD etiology. P or LP variants were found in patients A013, A016, A017, A022, A037, A041, A044, A050, A052, A054, A065, A075, A076, A078, A080, A082, A088, A096, A097, A100, A102, A105, A108, A110, A112, A118, A120, A121, A124, A125, A130 and A132.

### Whole exome sequence variants

A total of 381 variants were identified using WES, as summarized in Table 2. A summary of genes showing variants observed here is reported in Table 6.

**Table 6:** Summary of genes showing sequence variants

Gene	Variant (HGVS Coding)	Patients	Zygosity	Gene Function	GO Terms	Known ASD Gene (SFARI)	CADD Score
*AKAP9*	c.506 C > T	A013	Homozygous, Compound Heterozygous	Anchoring protein for PKA, regulates signaling pathways	GO:0005515 (protein binding), GO:0000165 (MAPK cascade)	Yes	29.3
*ABCA13*	c.4779_4780insGCGC	A052, A068	Compound Heterozygous	Involved in lipid transport and possibly neurological function	GO:0006810 (transport), GO:0030301 (cholesterol transport)	Yes	23.4
*AGBL4*	c.101G > T, c.-41 C > T	A071, A072, A073	Compound Heterozygous	Tubulin deglutamylase, involved in axonal transport and neuron development	GO:0098957 (anterograde axonal transport), GO:0021954 (CNS neuron development)	Yes	24.6, 11.7
*CIC*	c.1795 C > T	A070, A076	Compound Heterozygous, De novo	Transcriptional repressor involved in cell growth and development	GO:0003677 (DNA binding), GO:0045892 (negative regulation of transcription)	Yes	13.9
*DST*	c.2689 A > C	A052, A075	Compound Heterozygous	Cytoskeletal linker protein, critical for maintaining the structure of neural and epithelial cells	GO:0008092 (cytoskeletal protein binding), GO:0030155 (regulation of cell adhesion)	Yes	17.4
*EP400*	c.916 A > G	A110	Homozygous, Compound Heterozygous	Component of chromatin remodeling complex, involved in DNA repair and gene regulation	GO:0006338 (chromatin remodeling), GO:0006281 (DNA repair)	Yes	31.0
*EPPK1*	c.5101G > A	A038, A068	Compound Heterozygous	Cytoskeletal linker protein, involved in cytoskeletal architecture	GO:0005856 (cytoskeletal architecture)	No	24.8
*FAT1*	c.1505 A > G	A075	Compound Heterozygous	Cadherin involved in cell adhesion and cytoskeleton organization	GO:0007155 (cell adhesion), GO:0009897 (external side of plasma membrane)	Yes	13.2
*IL1RAPL1*	c.1507G > A	A118	Hemizygous	Involved in synapse formation and immune response regulation	GO:0006955 (immune response), GO:0050808 (synapse organization)	Yes	3.7
*KDM6B*	c.1415 A > G	A100, A102	Compound Heterozygous	Histone demethylase involved in gene expression and developmental processes	GO:0034720 (histone demethylation), GO:0045893 (positive regulation of transcription)	Yes	24.3
*LRP2*	c.682 C > A	A072	Compound Heterozygous	Multi-ligand endocytic receptor, involved in transport of nutrients and signaling molecules	GO:0005041 (low-density lipoprotein receptor activity), GO:0034389 (lipid transport)	Yes	34.0
*MAGEE2*	c.181G > A	A057, A058, A059	Hemizygous	Negative regulation of transcription by RNA polymerase II	GO:0000122 (negative regulation of transcription)	No	8.5
*NEXMIF*	c.700G > A	A044	Hemizygous	Involved in synaptic plasticity, learning, and memory	GO:0007399 (nervous system development), GO:0050890 (cognition)	Yes	22.9
*SYN1*	c.926G > A	A071	Hemizygous	Synapsin, involved in neurotransmitter release at synapses	GO:0007268 (synaptic transmission), GO:0007269 (neurotransmitter secretion)	Yes	10.9
*SYNE1*	c.1697 A > G	A096	Compound Heterozygous, De novo	Involved in nuclear anchoring and structural organization of the cytoskeleton	GO:0015629 (actin cytoskeleton), GO:0005884 (intermediate filament)	Yes	15.4
*TAF6*	c.14,185 C > T	A041	Homozygous, Compound Heterozygous	General transcription factor, part of TFIID complex involved in RNA polymerase II transcription	GO:0006366 (transcription from RNA polymerase II promoter), GO:0016607 (nuclear speck)	Yes	24.3
*KCND1*	c.1373G > A	A020	Hemizygous	Voltage-gated A-type potassium channel; membrane repolarization	GO:0005249 (voltage-gated potassium channel activity), GO:0071805 (potassium ion transport)	No	20.1
*PDE4DIP*	c.907G > C	A052, A094	Compound Heterozygous	Involved in microtubule assembly, Golgi apparatus organization, cell migration	GO:0030953 (microtubule assembly), GO:1,903,358 (Golgi apparatus organization), GO:0007098 (centrosome cycle)	No	20.2
*HMCN2*	c.2110 C > A	A075	Compound Heterozygous	Involved in synapse formation and neuronal development	GO:0007165 (signal transduction), GO:0035238 (neuron development)	Yes	18.5
*HIVEP3*	c.181G > A	A072	Hemizygous	Transcriptional activator involved in cell differentiation and proliferation	GO:0006355 (regulation of transcription), GO:0045664 (neuron differentiation)	No	15.8
*KIF13B*	c.126G > C	A071, A076	Compound Heterozygous	Motor protein involved in vesicular transport	GO:0030814 (vesicle-mediated transport), GO:0006886 (vesicle transport)	Yes	22.9
*RANBP17*	c.3045G > A	A037	Hemizygous	Involved in nuclear-cytoplasmic transport	GO:0006913 (nuclear transport), GO:0006606 (nuclear import)	Yes	19.3
*TAF1*	c.181G > A	A100	Compound Heterozygous	Transcription factor involved in the regulation of gene expression	GO:0006355 (regulation of transcription), GO:0000122 (negative regulation of transcription)	Yes	12.7
*ZNF462*	c.1903G > A	A063, A066	Compound Heterozygous	Zinc finger protein involved in transcription regulation	GO:0003677 (DNA binding), GO:0006396 (RNA processing)	No	25.5

Within the 317 non-synonymous coding variants, four were recurrent. For instance, the maternal hemizygous variant c.181G > A in the intronless gene *MAGEE2* was present in three siblings (A057, A058, A059). This variant was classified as VOUS (CADD score of 8.5). It is worthy of note that this variant is the unique recurrent variant among our cohort and segregates in the family (it is present in the mother and the maternal grandfather who showed autistic traits). *MAGEE2* encodes a member of the E subfamily of *MAGE* (melanoma antigen-encoding gene) family involved in the negative regulation of transcription by RNA polymerase II biological process (GO:0000122). Siblings A071, A072, and A073 carried two variants in compound heterozygosity in the *AGBL4* gene: c.101G > T (CADD score = 24.6) and c.41 C > T (CADD score = 11.7). Both variants were inherited from healthy parents. *AGBL4* is involved in biological processes such as anterograde and retrograde axonal transport of mitochondria (GO:0098957) and central nervous system neuron development (GO:0021954). Variant c.5101G > A in the *EPPK1* gene is recurrent in compound heterozygosity in two unrelated patients (A038 and A068). The encoded protein is involved in the organization of cytoskeletal architecture (GO:0005856).

Sequence variants were identified in 223 genes. Among them, 85 genes were already known to be associated with ASD in the SFARI database. Actually, 20 genes were recurrent (*ABCA13*, *AGBL4*, *AKAP9*, *CACNA1F*, *CDKL5*, *CIC*, *DDX53*, *DST*, *EP400*, *EPPK1*, *FAT1*, *IL1RAPL1*, *KDM6B*, *LRP2*, *NEXMIF*, *PLXNA3*, *PTK7*, *SYN1*, *SYNE1* and *TAF6*) and 138 genes were not included (six genes were recurrent: *KCND1*, *LOC101059915*, *MAGEE2*, *PDE4DIP*, *SOX3* and *SYTL4*). Therefore, a total of 27 genes were recurrent (see Table 6) and showed different zygosities among patients. Recurrent genes with homozygous variants were *AKAP9*, *EP400*, *LRP2*, *PTK7* and *TAF6*. *ABCA13*, *AGBL4*, *CIC*, *DST*, *EP400*, *EPPK1*, *FAT1*, *KDM6B*, *PDE4DIP*, *PTK7* and *SYNE1* showed variants in compound heterozygosity. Hemizygous variants were observed in *CACNA1F*, *CDKL5*, *DDX53*, *IL1RAPL1*, *KCND1*, *LOC101059915*, *MAGEE2*, *NEXMIF*, *PLXNA3*, *SOX3*, *SYN1* and *SYTL4*. Eventually, recurrent genes showing de novo heterozygous variants were *AKAP9*, *CIC*, *LRP2*, *SYNE1* and *TAF6*. Genes *KCND1* and *PDE4DIP* were noteworthy because of their function related to nervous system development or functional regulation, and the *MAGEE2* gene, since the variant was also present in other affected family members. *KCND1* showed two different hemizygous private variants in two patients: the splice site region variant c.1368 + 1G > A was found in patient A132 as its unique candidate variant and was classified as LP; the second variant is c.1373G > A in patient A020, classified as LB. The gene *KCND1* encodes a component of a membrane voltage-gated A-type potassium channel necessary for membrane repolarization. The activity of voltage-gated potassium channels (GO:0005249) is important in physiological processes such as the regulation of neurotransmitter release, heart rate, insulin secretion, and smooth muscle contraction and the gene provides potassium ions transmembrane transport (GO:0071805) and monoatomic ions transmembrane passage regulation (GO:0034765). *PDE4DIP* showed the compound heterozygous VOUS variants c.907G > C and c.6905 A > T in patient A052 and c.1546 C > T and c.5341 C > T in A094. *PDE4DIP* encodes a protein involved in microtubule assembly and nucleation (GO:0030953- GO:0090063), in the regulation of Golgi apparatus organization (GO:1903358) and in the centrosome cycle (GO:0007098), contributing to cell migration, mitotic spindle orientation and cell-cycle progression. Moreover, A052 showed two additional variants in compound heterozygosity: c.2380G > A and c.1399G > C in the *HMCN2* gene encoding a protein with involvement in cell adhesion mediated by integrins (GO:0007155) and hemostasis regulation (GO:1900047). The variant c.2380G > A has been classified as LP and c.1399G > C as LB. Patient A094 showed a second compound heterozygosity in the *TRPM2* gene: c.52G > A and c.4654G > A, both classified as LB. *TRPM2* is a non-specific cation channel that mediates calcium influx (GO:0005262).

Ninety-five private variants (*i.e.*, unique variants) in 89 genes were identified in 61 patients. Among them, two were pathogenic (2.1% in the sample of private variants), 24 LP (25.3%), 61 VOUS (64.2%), and eight likely benign (8.4%). Among the identified private pathogenic variants, A132 showed the hemizygous c.1368 + 1G > A variant in the *KCND1* gene, inherited from his healthy mother, as its unique selected candidate variant. This variant is a splice site alteration with predicted loss of function of the gene product. It has been classified as LP (CADD score = 34), but, considering that it is the only genetic candidate and the presence of neurodevelopmental phenotypes in other patients with variants in the *KCND1* gene, it can be classified as pathogenic. The gene product is part of the voltage-gated A-type potassium channels (GO:0034765) and has functional relevance for potassium ions transmembrane transport (GO:0071805) in neurons and for the regulation of neurotransmitter release and membrane repolarization after action potential. The gene has already been included in the SFARI genes database as 2B gene (Strong candidate). The remaining 24 LP variants are mostly compound heterozygous, with three homozygous variants, two *de novo* heterozygous and two hemizygous.

### Copy number variants

A short summary of CNVs observed here is reported in Tables 3 and 7. Parental transmission was equally distributed between paternal and maternal sources.

**Table 7:** Copy number variants, classification, encompassed genes and rearrangement

CNV - ISCN 2020	Inheritance	Size	CNV classification	Genes	Genes of Interest	SFARI Score	Type of gene rearrangement
13q14.13q14.2(47240527_48096722)x1	pat	856.2 kb	VOUS	*LRCH1*			INT START
				*ESD*			DELETED
				*HTR2A*	*HTR2A*		DELETED
13q12.11(20146801_20270834)x3	pat	124.03 kb	VOUS	*MPHOSPH8*	*MPHOSPH8*		DUPLICATED
				*PSPC1*			INT END
13q12.11(20411945_20559030)x3	pat	147.09 kb	VOUS	*ZMYM5*	*ZMYM5*		INT START
				*ZMYM2*	*ZMYM2*	S	INT END
17q21.31(41409849_41597925)x3	mat	188.08 kb	VOUS	*DHX8*	*DHX8*		INT END
7q11.22(69168540_69198438)x1	mat	29.9 kb	LP	*AUTS2*	*AUTS2*	1	INTRAGENIC
16p13.11(14968878_16311041)x1	de novo	1.34 Mb	LP	*NOMO1*			INT START
				*NPIPA1*			DELETED
				*PDXDC1*			DELETED
				*NTAN1*			DELETED
				*RRN3*			DELETED
				*MPV17L*			DELETED
				*MARF1*			DELETED
				*NDE1*	*NDE1*		DELETED
				*MYH11*			DELETED
				*FOPNL*			DELETED
				*ABCC1*			DELETED
				*ABCC6*			INT END
16q21(60064996_63555913)x1	mat	3.49 Mb	LP	*CDH8*	*CDH8*	2	DELETED
3q24(147111840_147118109)x3	de novo	8.91 kb	VOUS	*ZIC1*	*ZIC1*		INTRAGENIC
				*ZIC4*			INTRAGENIC
15q13.1(29656854_29783953)x1	pat	127.1 kb	VOUS	*FAM189A1*	*FAM189A1*		INTRAGENIC
Xp22.33(426261_679415)x2	mat	253.16 kb	LP	*SHOX*	*SHOX*	2	DUPLICATED
16q23.3(82184809_83663330)x3	mat	1.48 Mb	VOUS	*CDH13*	*CDH13*	2	INT END
				*MPHOSPH6*			INT START
7q21.11(81472446_81639364)x3	pat	166.92 kb	VOUS	*CACNA2D1*	*CACNA2D1*	2	INT END
16q23.2(80751184_80928051)x1	mat	176.87 kb	VOUS	*CDYL2*	*CDYL2*		INT START
Xq21.1(77081624_77139044)x2	mat	57.42 kb	VOUS	*MAGT1*	*MAGT1*		INT END
1q21.1q21.2(146542653_147872312)x1	pat	1.33 Mb	LP	*PRKAB2*			DELETED
				*FMO5*			DELETED
				*BCL9*			DELETED
				*CHD1L*			DELETED
				*ACP6*			DELETED
				*GJA5*			DELETED
				*GJA8*			DELETED
				*GPR89B*			DELETED
1q21.1q21.2(146499008_147860611)x3	pat	1.36 Mb	LP	*PRKAB2*			DUPLICATED
				*FMO5*			DUPLICATED
				*BCL9*			DUPLICATED
				*CHD1L*			DUPLICATED
				*ACP6*			DUPLICATED
				*GJA5*			DUPLICATED
				*GJA8*			DUPLICATED
				*GPR89B*			DUPLICATED
6q22.31(124068036_124449980)x3	mat	382 kb	VOUS	*NKAIN2*	*NKAIN2*		INT END
11q25(133124721_134353783)x3	mat	1.23 Mb	VOUS	*OPCML*			INT START
				*SPATA19*			DUPLICATED
				*IGSF9B*			DUPLICATED
				*JAM3*			DUPLICATED
				*NCAPD3*			DUPLICATED
				*VPS26B*			DUPLICATED
				*THYN1*			DUPLICATED
				*ACAD8*			DUPLICATED
				*B3GAT1*			DUPLICATED
Yp11.32(1421350_1634267)x2	pat	212.9 kb	VOUS	*IL3RA*			INT START
				*SLC25A6*			DUPLICATED
				*ASMTL*			DUPLICATED
				*P2RY8*			DUPLICATED
9q33.1(119387004_119527204)x1	mat	140.2 kb	LP	*TRIM32*	*TRIM32*	3	DELETED
				*ASTN2*	*ASTN2*	2	INTRAGENIC
10q23.1(84137861_84181161)x1	pat	43.3 kb	VOUS	*NRG3*	*NRG3*		INTRAGENIC
16q24.3(89428111_89453232)x3	pat	25.12 kb	VOUS	*ANKRD11*	*ANKRD11*	1	INTRAGENIC
5q11.2(51915987_53470988)x1	mat	1.56 Mb	VOUS	*PELO*			DELETED
				*ITGA1*			DELETED
				*ITGA2*			DELETED
				*MOCS2*			DELETED
				*FST*			DELETED
				*NDUFS4*			DELETED
				*ARL15*			DELETED
6q27(165421392_165801341)x3	pat	335.6 kb	VOUS	*PDE10A*	*PDE10A*		INT END
1p32.1(59947236_60012581)x1	mat	65.35 kb	VOUS	*FGGY*	*FGGY*		INTRAGENIC
6p21.32(32179815_32312148)x1	mat	132.33 kb	VOUS	*NOTCH4*	*NOTCH4*		DELETED
11q14.1(84455989_84607402)x1	mat	151.4 kb	LP	*DLG2*	*DLG2*	2	INTRAGENIC
15q11.2(25226260_25241828)x3	mat	15.57 kb	VOUS	*SNURF*	*SNURF*		INTRAGENIC
				*SNHG14*			INTRAGENIC
16p13.3(7041435_7132676)x1	pat	91.24 kb	LP	*RBFOX1*	*RBFOX1*	2	INTRAGENIC
Xp22.33(1189595_1383266)x2 Yp11.32(1162459_1322464)x2	?	192.42 kb	VOUS	*CRLF2*	*CRLF2*		DUPLICATED
7q36.3(158801633_158903310)x3	mat	101.68 kb	VOUS	*VIPR2*	*VIPR2*		INT END
Xp22.31(7532513_8115193)x2	mat	582.68 kb	VOUS	*VCX*			DUPLICATED
				*PNPLA4*			DUPLICATED
Xp22.33(169791_537202)x2	mat	367.41 kb	VOUS	*PLCXD1*			DUPLICATED
				*GTPBP6*			DUPLICATED
				*PPP2R3B*			DUPLICATED
3q29(196418238_196487740)x3	pat	69.5 kb	VOUS	*PIGX*			INT START
				*PAK2*	*PAK2*	2	INT END
13q14.11(43558077_43731680)x3	mat	173.6 kb	VOUS	*EPSTI1*			INT START
				*DNAJC15*			DUPLICATED
2q23.1(148805626_148936476)x1	mat	130.85 kb	LP	*MBD5*	*MBD5*	1	INTRAGENIC
13q12.11(20413829_20445336)x3	mat	31.51 kb	VOUS	*ZMYM5*	*ZMYM5*		INT START
Yp11.32(771993_1322464)x2	pat	550 kb	VOUS	*CRLF2*	*CRLF2*		DUPLICATED
8q24.3(144825929_144881529)x3	pat	55.6 kb	VOUS	*SCRIB*	*SCRIB*		INT END
14q31.1(79388339_79657573)x1	de novo	269.24 kb	P	*NRXN3*	*NRXN3*	1	INTRAGENIC
13q32.2(99177988_99271292)x3	pat	93.31 kb	VOUS	*STK24*	*STK24*		INT START
4q25(110390080_110653618)x3	mat	263.54 kb	VOUS	*SEC24B*	*SEC24B*		INT START
				*CASP6*			DUPLICATED
				*PLA2G12A*			DUPLICATED
4q25(113167151_113610549)x3	mat	443.4 kb	VOUS	*AP1AR*	*AP1AR*		INT START
				*TIFA*			DUPLICATED
				*ALPK1*			DUPLICATED
				*NEUROG2*	*NEUROG2*		DUPLICATED
1q21.1q21.2(145804790_147860611)x3	pat	2.06 Mb	LP	*GPR89A*			INT START
				*NBPF11*			DUPLICATED
				*HYDIN2*	*HYDIN2*	2	DUPLICATED
				*NBPF12*			DUPLICATED
				*PRKAB2*			DUPLICATED
				*FMO5*			DUPLICATED
				*BCL9*			DUPLICATED
				*CHD1L*			DUPLICATED
				*ACP6*			DUPLICATED
				*GJA5*			DUPLICATED
				*GJA8*			DUPLICATED
				*GPR89B*			DUPLICATED
12q24.12q24.13(112184121_112302954)x3	pat	118.83 kb	VOUS	*ACAD10*			INT START
				*ALDH2*			DUPLICATED
				*MAPKAPK5*			INT END
Xq12(65768203_65926180)x2	mat	155.7 kb	VOUS	*EDA2R*	*EDA2R*		DUPLICATED

A total of 46 CNVs were identified in 35 out of 122 patients (28.68%). No differences in ratios between males and females were observed: 28.9% of males (30 out of 104) and 27.8% of females (5 out of 18) tested positive for array-CGH. Among the CNVs, 16 deletions (34.8%) and 30 duplications (65.2%) were found. A total of 11 CNVs (8 deletions and 3 duplications) were intragenic (24%). When considering pathogenic and likely pathogenic CNVs, deletions were more frequent, with 9 deletions and 3 duplications. In the VOUS CNVs subgroup, 7 deletions and 27 duplications were identified, totaling 34 CNVs. One *de novo* CNV was found in cytoband 14q31.1 in patient A120 (0.82% of patients). The 14q31.1 microdeletion, classified as pathogenic, resulted in an intragenic deletion in the *NRXN3* gene (MIM *600567). Diseases associated with *NRXN3* mutations include ASD. *NRXN3* is involved in neuronal cell adhesion, axon guidance, learning, and social behavior. This CNV may be considered a candidate variant for Essential ASD. Annunziata et al. [11] found 3.1% of patients with pathogenic CNVs in an Essential Autism cohort, while Pinto et al. [26] found pathogenic rearrangements in 2.8% of non-syndromic autistic patients. Noticeably, the frequency of pathogenic CNVs in essential ASD patients is significantly lower than in complex ASD patients (estimated around 10%) [6].

In this study, 11 likely pathogenic CNVs were found in 11 patients (9% of the total). Ten CNVs were inherited from a healthy parent (six maternal, four paternal); one CNV had a *de novo* origin. The CNVs were ranked by chromosome: 1q21.1q21.2 paternal deletion in patient A050, 1q21.1q21.2 paternal duplication in patients A054 and A124, 2q23.1 maternal deletion in patient A110, 7q11.22 maternal deletion in patient A013, 9q33.1 maternal deletion in patient A065, 11q14.1 maternal deletion in patient A080, 16p13.11 *de novo* deletion in patient A016, 16q21 maternal deletion in patient A017, 16p13.3 paternal deletion in patient A082, and Xp22.33 maternal duplication in patient A022.

A total of 34 CNVs were classified as VOUS in 27 ASD patients (22%). The co-occurrence of a likely pathogenic CNV and a VOUS CNV was observed in four patients (4% of the cohort). Pathogenic and likely pathogenic CNVs in 12 out of 133 essential ASD patients (9%) were previously reported by Napoli et al. [2]. Discovering P and LP CNVs in 12 patients out of 122, the results are overlapping (9.8% in our cohort). A 1q21.1q21.2 microdeletion in one patient suggests a predisposing factor for Essential Autism. The 1q21.1q21.2 reciprocal microduplication was found in two patients. This duplication is the unique recurrent CNV classified as LP in the cohort. Overlapping genes include *PRKAB2*, *FMO5*, *BCL9*, *CHD1L*, *ACP6*, *GJA5*, *GJA8*, and *GPR89B*. The 1q21.1q21.2 microduplication and reciprocal microdeletion show the same phenotype as reported in the literature. The prevalence in developmental delay and intellectual disability patients is 0.12%, compared to 0.2% for the reciprocal microdeletion. Reduced penetrance and variable expressivity were reported [27]. The intragenic microdeletion in the *MBD5* gene (2q23.1) found in patient A110 could be considered a susceptibility factor for essential ASD. Deletions in this gene were reported in 0.18% of cases as responsible for autism and other neurodevelopmental diseases [28]. About 90% of individuals with *MBD5* haploinsufficiency show a *de novo* 2q23.1 microdeletion. Additionally, partial and complete *MBD5* microdeletions were inherited from a mildly affected parent [29, 30]. *MBD5* is involved in nervous system development, regulation of behavior, and regulation of multicellular organism growth. The 2q23.1 microdeletion in the cohort is inherited from an apparently healthy mother, supporting incomplete penetrance.

The 7q11.22 deletion (patient A013) is an intragenic deletion in *AUTS2*, reported in the SFARI database (SFARI score = 1). This gene is known to be expressed in the brain and involved in neurodevelopmental disorders including ASD. *AUTS2* plays a role in axon and dendrite extension, neuron migration, and actin cytoskeleton reorganization. Most pathogenic variants reported to date are *de novo* intragenic deletions [31], but inherited *AUTS2* rearrangements are also reported [32]. In the analyzed patient, the same *AUTS2* deletion is present in the healthy mother and grandmother. The 9q33.1 microdeletion (patient A065) causes the deletion of the *TRIM32* gene and the intragenic deletion of *ASTN2*. Disruptions or deletions of *TRIM32* are more frequent in male patients with Neurodevelopmental Disease (most common diagnoses: Autism, ADHD, speech-language delay). *TRIM32* is highly expressed in the brain during early prenatal development, particularly in the cerebellar cortex [33]. It is involved in neurogenesis and neuron differentiation. Deletions or disruptions of *ASTN2* are significantly enriched in male subjects with neurodevelopmental defects, with known reduced penetrance. *ASTN2* is involved in neuron cell-cell adhesion and migration. A microdeletion in 11q14.1 found in patient A080 interrupted the *DLG2* gene, reported in the SFARI database. *DLG2* is involved in axonal protein transport, chemical synaptic transmission, receptor localization to synapse, and synaptic stability at cholinergic synapses. *DLG2* deficiency induces autism-related behavioral phenotypes [34–36]. A *de novo* 16p13.11 microdeletion in patient A016 predisposes to cognitive impairment, autism, seizures, and microcephaly. This variant shows variable expression and incomplete penetrance [37, 38].

The *CDH8* gene was completely deleted in the 16q21 microdeletion (patient A017). *CDH8* haploinsufficiency is an autism and intellectual disability susceptibility factor, playing a key role in cerebellar development. Pathogenic variants in *CDH8* cause overgrowth diseases [39, 40]. A 16p13.3 microdeletion in patient A082 involved the *RBFOX1* gene, reported in the SFARI database. *RBFOX1* haploinsufficiency causes neurodevelopmental phenotypes including autism, intellectual disability, and epilepsy. Inherited *RBFOX1* variants from healthy parents raise doubts about *RBFOX1* CNVs pathogenicity [41–43]. An Xq21.1 duplication found in patient A045 is a novel finding in ASD patients, with no prior reports concerning autism. The CNV is maternally inherited, suggesting possible hemizygosity effects. The recurrent CNVs found (see Supplementary Table 2, Genome build GRCh37/hg19) differ from those reported by Annunziata et al. [11]: 2p16.3 microdeletion in two patients and 15q11.2 microduplication in two patients. The recurrent CNVs in this cohort were the 1q21.1q21.2 microduplication and two microduplications, 13q12.11 (patients A006 and A113) and Yp11.32 (patients A088 and A119), which are not reported in the literature as associated with ASD. Despite their recurrence, the genes in these rearrangements do not appear central to ASD pathogenesis.

## Conclusions

Our study’s comprehensive genetic analysis revealed a high prevalence of genetic variants detected through WES and array-CGH. We identified a significant number of pathogenic and likely pathogenic variants, emphasizing the importance of thorough genetic testing in understanding the etiology of autism spectrum disorders. The overall detection rate for pathogenic variants was comparable to or slightly lower than previously reported rates in the literature, while the inclusion of LP variants significantly increased the DR, suggesting their potential clinical relevance.

### Pathogenic and LP variants

Our findings highlighted several recurrent genes and variants, suggesting potential new susceptibility factors for ASD. Variants in genes such as *MAGEE2*, *AGBL4*, and *EPPK1*, among others, were recurrent and involved in biological processes critical for neuron differentiation, axonal transport, and cytoskeletal organization.

### Copy number variants (CNVs)

We detected CNVs in a significant portion of our cohort, including both deletions and duplications. Pathogenic and LP CNVs were identified in several genomic regions containing genes implicated in neurodevelopmental and synaptic functions. VOUS CNVs also revealed potentially significant genetic alterations requiring further investigation.

### Clinical implications

The study’s results underscore the utility of combining WES and array-CGH in detecting genetic variants associated with ASD. The high number of variants of uncertain significance (VOUS) suggests the need for functional studies to elucidate their roles. Furthermore, the identification of recurrent variants and genes provides insights into potential genetic mechanisms underlying ASD, contributing to the growing body of knowledge necessary for improved diagnostic and therapeutic strategies. As a limitation, non-coding variants and regulation of genes were not considered, even though non-coding regions could account for a significant percentage of the ASD genetic diagnosis [44].

### Future directions

Future research should focus on expanding cohort sizes and conducting functional studies to validate the clinical relevance of identified variants. Larger cohorts will help confirm new susceptibility genes and improve our understanding of ASD’s genetic architecture, ultimately aiding in the development of more targeted and effective interventions for affected individuals. Moreover, the landscape of DNA sequencing is continuously advancing, with new players and techniques to decode genetic information emerging, including the entire Genome Sequencing with short and long reads, which will provide a marginal, but important, increase in diagnostic yield for ASD patients.

## Electronic supplementary material

Below is the link to the electronic supplementary material.

Supplementary Material 1

Supplementary Material 2

## Data Availability

All data are available on European Variation Archive: https://www.ebi.ac.uk/eva/?Study-Browser&browserType=sgv. Project: PRJEB81859. Analyses: ERZ24903986.
