# Supplementary material for: Unveiling genetic insights: Array-CGH and WES discoveries in a cohort of 122 children with essential autism spectrum disorder
Source: BMC Genomics. 2024 Dec 10;25:1186. doi: 10.1186/s12864-024-11077-5 (PMC11629504; doi:10.1186/s12864-024-11077-5)
Supplement: Supplementary file 1 — Supplementary Material 1 [file 12864_2024_11077_MOESM1_ESM.pdf]

Supplementary Table 1. Sequence variants identified by whole exome sequencing.

CASE	SEX	GENE	SFARI SC	GENETIC STATE	HGVSC	HGVSP	LOCATION	Max AF	CADD score	ACMG	VARIANT CLASSIFICATION WES
A001	F	OTOP3	//	COMP HET	c.17G>C	p.Arg6Pro	17:72931913	0	23	UncertainSignificance	VOUS
A001	F	OTOP3	//	COMP HET	c.154G>A	p.Val52Met	17:72937568	0,000233155	0,001	UncertainSignificance	VOUS
A001	F	ACRBP	//	COMP HET	c.1487delT	p.Leu496fs	12:6748141	0	33	UncertainSignificance	VOUS
A001	F	ACRBP	//	COMP HET	c.431T>C	p.Val144Ala	12:6754430	8,13253E-06	5,049	UncertainSignificance	VOUS
A001	F	IQCN	//	COMP HET	c.3332A>G	p.His1111Arg	19:18368201	0,001162791	0,063	UncertainSignificance	VOUS
A001	F	IQCN	//	COMP HET	c.349T>C	p.Tyr117His	19:18378001	0	10,7	UncertainSignificance	VOUS
A001	F	B3GNT8	//	COMP HET	c.557C>T	p.Ser186Phe	19:41932127	0,003372093	25.8	UncertainSignificance	VOUS
A001	F	B3GNT8	//	COMP HET	c.210T>G	p.Phe70Leu	19:41932474	0,001981352	0.369	UncertainSignificance	VOUS
A001	F	AKAP9	2	HOM	c.10459G>A	p.Glu3487Lys	7:91726960	0,000813953	26.3	UncertainSignificance	VOUS
A002	M	PPP2R1B	2	COMP HET	c.1645C>T	p.Arg549Cys	11:111613299	0,00013006	34	LikelyPathogenic	P
A002	M	PPP2R1B	2	COMP HET	c.269G>A	p.Gly90Asp	11:111635566	0,012217826	15,11	UncertainSignificance	LB
A002	M	GPRASP1	//	HEM	c.3535A>G	p.Met1179Val	X:101912376	3,91578E-05	23.2	UncertainSignificance	LP
A003	M	ATRX	1	HEM	c.2265T>G	p.Asn755Lys	X:76938483	0	18,73	UncertainSignificance	VOUS
A003	M	SOX3	//	HEM	c.571G>A	p.Glu191Lys	X:139586655	0	32	UncertainSignificance	VOUS
A005	F	PTK7	1	COMP HET	c.86C>A	p.Pro29His	6:43044707	0,003369587	12.95	LikelyBenign	LB
A005	F	PTK7	1	COMP HET	c.1085G>A	p.Arg362Gln	6:43100282	0,00016275	16.86	UncertainSignificance	VOUS
A005	F	FAT1	2	COMP HET	c.6782C>T	p.Thr2261Met	4:187540958	0,000998403	24.3	UncertainSignificance	VOUS
A005	F	FAT1	2	COMP HET	c.2563G>A	p.Gly855Arg	4:187628419	0,003128008	24.2	UncertainSignificance	VOUS
A006	M	MAGEC1	//	HEM	c.1950T>G	p.Ser650Arg	X:140995140	0,000148633	0.251	UncertainSignificance	VOUS
A006	M	RBMXL3	//	HEM	c.1108C>T	p.Arg370Cys	X:114425112	1,83719E-05	18.52	UncertainSignificance	VOUS
A006	M	FATE1	//	HEM	c.298G>C	p.Glu100Gln	X:150889930	0,000445898	21.1	UncertainSignificance	VOUS
A007	M	CAPN9	//	COMP HET	c.655_656insT	p.Leu220fs	1:230903405	0	28	UncertainSignificance	VOUS
A007	M	CAPN9	//	COMP HET	c.881C>T	p.Pro294Leu	1:230910305	0,000226963	14.04	UncertainSignificance	LB
A007	M	CT83	//	HEM	c.313G>A	p.Gly105Ser	X:115592937	5.46e-6	8.441	UncertainSignificance	LB
A007	M	LOC101059915	//	HEM	c.97C>T	p.Arg33Trp	X:70887750	0,000264901	2.851	UncertainSignificance	LB
A007	M	MMACHC	//	COMP HET	c.276G>T	p.Glu92Asp	1:45973222	1,62555E-05	23.5	UncertainSignificance	VOUS
A007	M	MMACHC	//	COMP HET	c.818G>C	p.Arg273Thr	1:45974856	0	1.436	UncertainSignificance	LB
A008	M	AHDC1	1	COMP HET	c.4711C>T	p.Pro1571Ser	1:27873916	4,56429E-06	23.8	UncertainSignificance	VOUS
A008	M	AHDC1	1	COMP HET	c.4000G>A	p.Gly1334Ser	1:27874627	0,000599042	10.97	LikelyBenign	LB
A008	M	B4GALT2	//	COMP HET	c.174_175insAGCAGC	p.Ser59_Ser60insSerSer	1:44447006	4,66699E-05	15.9	UncertainSignificance	VOUS
A008	M	B4GALT2	//	COMP HET	c.605C>T	p.Ala202Val	1:44450592	5,68791E-05	23.4	UncertainSignificance	VOUS
A008	M	PTPRF	//	COMP HET	c.635C>T	p.Ser212Leu	1:44044547	8,124E-06	27.2	UncertainSignificance	VOUS
A008	M	PTPRF	//	COMP HET	c.2698T>A	p.Leu900Met	1:44069521	4,06306E-06	18.77	UncertainSignificance	VOUS
A008	M	KLF8	//	HEM	c.72_73insT	p.Lys26fs	X:56276728	5,64137E-06	23.2	UncertainSignificance	VOUS
A008	M	SLC35A2	//	HEM	c.1021C>G	p.Pro341Ala	X:48762165	5,66187E-06	23.8	UncertainSignificance	VOUS
A009	M	CC2D1A	2	COMP HET	c.-191G>T		19:14017064	0	6.163	UncertainSignificance	VOUS
A009	M	CC2D1A	2	COMP HET	c.566C>T	p.Ala189Val	19:14024269	0,011981788	0.576	LikelyBenign	LB
A009	M	NSUN2	//	COMP HET	c.254+1G>A		5:6632711	0	33	Pathogenic	LP
A009	M	NSUN2	//	COMP HET	c.-73C>T		5:6633165	0,0115815	6.54	Benign	LB
A010	M	CACNA1F	2	HEM	c.5474G>A	p.Arg1825His	X:49063003	0,000148633	22.2	UncertainSignificance	VOUS
A010	M	PLXNB3	//	HEM	c.3289G>A	p.Val1097Ile	X:153039323	0,000148655	11.37	UncertainSignificance	VOUS
A010	M	ASB11	//	HEM	c.295G>C	p.Val99Leu	X:15315770	5,6803E-05	26.1	UncertainSignificance	VOUS
A011	M	CACNA1G	2	COMP HET	c.354+5_354+6delGT		17:48646346	0	15.34	UncertainSignificance	VOUS
A011	M	CACNA1G	2	COMP HET	c.3000A>C	p.Glu1000Asp	17:48673943	0,000199681	15.27	UncertainSignificance	VOUS
A011	M	L3MBTL1	//	COMP HET	c.947G>A	p.Trp316*	20:42159462	0,000191197	42	UncertainSignificance	VOUS
A011	M	L3MBTL1	//	COMP HET	c.1627A>G	p.Arg543Gly	20:42164548	6,78372E-05	25.0	LikelyBenign	VOUS
A011	M	STARD9	//	COMP HET	c.158A>G	p.Lys53Arg	15:42877759	0,001572327	24.9	UncertainSignificance	VOUS
A011	M	STARD9	//	COMP HET	c.8410G>T	p.Ala2804Ser	15:42982186	9,80049E-05	2.621	UncertainSignificance	LB
A011	M	DHODH	//	COMP HET	c.833G>A	p.Gly278Glu	16:72057077	8,04305E-06	26.4	UncertainSignificance	VOUS
A011	M	DHODH	//	COMP HET	c.982C>A	p.Pro328Thr	16:72057381	4,00917E-06	24.7	UncertainSignificance	VOUS

A011	M	MAML1	//	COMP HET	c.2002C>T	p.Arg668Cys	5:179198178	0,000116279	32	UncertainSignificance	VOUS
A011	M	MAML1	//	COMP HET	c.2003G>A	p.Arg668His	5:179198179	7,95469E-06	32	UncertainSignificance	VOUS
A011	M	NRXN1	1	HETdn	c.-117C>T		2:50201255	0,003311047	21.5	UncertainSignificance	VOUS
A012	M	UPF3B	1	HEM	c.931C>T	p.Arg311Cys	X:118972367	2,24006E-05	26.8	UncertainSignificance	VOUS
A012	M	RANGAP1	//	COMP HET	c.1152_1154delGGA	p.Glu385del	22:41650417	0,001574994	12.87	UncertainSignificance	LB
A012	M	RANGAP1	//	COMP HET	c.502G>A	p.Glu168Lys	22:41657563	0,000199681	24.0	UncertainSignificance	VOUS
A013	M	PGK1	//	HEM	c.506C>T	p.Ala169Val	X:77372897	0,001	29.3	LikelyPathogenic	LP
A013	M	CIC	1	HETdn	c.4779_4780insGCGC	p.Pro1594fs	19:42799295	0	23.4	UncertainSignificance	LP
A013	M	FBXO33	2	COMP HET	c.101_109delAGCTGCGAC	p.Gln34_Arg36del	14:39901257	0,006839526	20.2	LikelyBenign	VOUS
A013	M	FBXO33	2	COMP HET	c.16T>A	p.Ser6Thr	14:39901351	0,000206889	25.0	UncertainSignificance	VOUS
A013	M	SLC12A3	//	COMP HET	c.37G>C	p.Ala13Pro	16:56899184	0,001395349	3.409	UncertainSignificance	LB
A013	M	SLC12A3	//	COMP HET	c.1795C>T	p.Leu599Phe	16:56918086	8,56046E-05	25.3	UncertainSignificance	VOUS
A014	M	EGFL6	//	HEM	c.1551+1G>C		X:13645396	0	23.7	UncertainSignificance	VOUS
A014	M	PTPRB	2	COMP HET	c.1898G>A	p.Arg633Gln	12:70974842	0,000241371	32	UncertainSignificance	VOUS
A014	M	PTPRB	2	COMP HET	c.499G>C	p.Glu167Gln	12:70989934	0	3,72	UncertainSignificance	VOUS
A014	M	HIVEP2	1	COMP HET	c.3383C>T	p.Pro1128Leu	6:143092493	0,000199681	16,67	UncertainSignificance	VOUS
A014	M	HIVEP2	1	COMP HET	c.1471A>T	p.Ser491Cys	6:143094405	0	10.19	UncertainSignificance	VOUS
A014	M	VEGFD	//	HEM	c.116G>C	p.Arg39Pro	X:15381416	0	16.47	UncertainSignificance	VOUS
A014	M	MBTPS2	//	HEM	c.706A>G	p.Ile236Val	X:21886620	0,000148633	19.20	UncertainSignificance	VOUS
A015	F	USH2A	2	COMP HET	c.15095G>A	p.Ser5032Asn	1:215808003	0,001	7,243	UncertainSignificance	VOUS
A015	F	USH2A	2	COMP HET	c.8681+6A>T		1:216051094	0	17,66	UncertainSignificance	VOUS
A015	F	USH2A	2	COMP HET	c.6628C>G	p.Pro2210Ala	1:216172258	0,012	19,41	LikelyBenign	VOUS
A016	M	ABCC1	//	HET	c.3901C>T	p.Arg1301Cys	16:16225727	0,000827973	25,2	UncertainSignificance	VOUS
A019	M	TCEAL2	//	HEM	c.78C>G	p.His26Gln	X:101381880	0	6.371	UncertainSignificance	LB
A019	M	GRIN2A	1	COMP HET	c.4307A>G	p.Asn1436Ser	16:9857094	0,000618047	16.74	UncertainSignificance	VOUS
A019	M	GRIN2A	1	COMP HET	c.2899G>C	p.Val967Leu	16:9858502	0,007674419	15.90	Benign	LB
A020	M	KCND1	//	HEM	c.1373G>A	p.Ser458Asn	X:48823079	0	9.926	UncertainSignificance	LB
A020	M	SPEN	2	COMP HET	c.9730A>C	p.Thr3244Pro	1:16262465	0,039960725	3.812	Benign	LB
A020	M	SPEN	2	COMP HET	c.10391G>C	p.Gly3464Ala	1:16264022	0,00255814	22.7	UncertainSignificance	VOUS
A021	F	LRP2	2	HOM	c.13803G>A	p.Met4601Ile	2:169985338	0,00771675	23.4	LikelyBenign	VOUS
A021	F	MS4A18	//	COMP HET	c.314C>T	p.Thr105Met	11:60497102	6,95185E-05	15,44	UncertainSignificance	VOUS
A021	F	MS4A18	//	COMP HET	c.330G>A	p.Trp110*	11:60497118	0	26,7	UncertainSignificance	VOUS
A021	F	YLPM1	//	COMP HET	c.4873A>G	p.Met1625Val	14:75276434	0,000120337	16,12	UncertainSignificance	VOUS
A021	F	YLPM1	//	COMP HET	c.5903G>T	p.Gly1968Val	14:75283937	2,22902E-05	24,6	UncertainSignificance	VOUS
A021	F	CIC	1	COMP HET	c.419G>A	p.Arg140Gln	19:42776354	0	6,992	UncertainSignificance	VOUS
A021	F	CIC	1	COMP HET	c.157G>T	p.Val53Leu	19:42791012	0	22,8	UncertainSignificance	VOUS
A022	M	AMER1	//	HEM	c.1255C>T	p.Pro419Ser	X:63411912	0	7.174	LikelyBenign	LB
A022	M	NHS	//	HEM	c.1100C>T	p.Ser367Leu	X:17742473	0,000297265	25.1	UncertainSignificance	VOUS
A022	M	CXorf67	//	HEM	c.836G>A	p.Arg279His	X:51150704	4,14267E-05	0.014	UncertainSignificance	VOUS
A024	M	RPL3L	//	HOM	c.280G>A	p.Ala94Thr	16:2002960	0,006860465	21.0	UncertainSignificance	VOUS
A024	M	DST	2	COMP HET	c.5909T>C	p.Phe1970Ser	6:56482923	0,004464286	20.6	LikelyBenign	VOUS
A024	M	DST	2	COMP HET	c.3130A>G	p.Met1044Val	6:56490044	2,43748E-05	26.4	UncertainSignificance	VOUS
A024	M	SEC31B	//	COMP HET	c.1759G>T	p.Glu587*	10:102257890	0,000581395	37	UncertainSignificance	VOUS
A024	M	SEC31B	//	COMP HET	c.323C>T	p.Ser108Leu	10:102269149	0,000348837	21.8	UncertainSignificance	VOUS
A024	M	PDZD2	//	COMP HET	c.4528G>A	p.Asp1510Asn	5:32088082	0,00068089	19,87	UncertainSignificance	VOUS
A024	M	PDZD2	//	COMP HET	c.5508G>T	p.Met1836Ile	5:32089062	0	0,636	UncertainSignificance	VOUS
A025	M	PREX2	//	COMP HET	c.2648G>A	p.Ser883Asn	8:69012011	0	21.7	UncertainSignificance	VOUS
A025	M	PREX2	//	COMP HET	c.3644G>A	p.Arg1215Gln	8:69033204	0,000998403	22.8	UncertainSignificance	VOUS
A025	M	DHX8	//	HETdn	c.2534C>G	p.Ser845Trp	17:41590761	0	33	UncertainSignificance	VOUS
A026	M	SYN1	1	HEM	c.1615G>A	p.Gly539Ser	X:47433768	0,000533618	20.1	UncertainSignificance	VOUS
A026	M	ACTL9	//	HOM	c.1199G>A	p.Arg400Gln	19:8807853	0,000453926	23.5	UncertainSignificance	VOUS
A026	M	OR2Z1	//	HOM	c.473T>A	p.Ile158Asn	19:8841863	0,000798722	22.8	UncertainSignificance	VOUS
A026	M	OR2Z1	//	HOM	c.653A>G	p.Tyr218Cys	19:8842043	0,000798722	24.4	UncertainSignificance	VOUS

A026	M	ZHX1	//	COMP HET	c.1325C>T	p.Pro442Leu	8:124266862	0,000465116	17.47	UncertainSignificance	VOUS
A026	M	ZHX1	//	COMP HET	c.376C>A	p.Pro126Thr	8:124267811	0,000199681	22.9	UncertainSignificance	VOUS
A026	M	RBMXL3	//	HEM	c.1673G>A	p.Arg558His	X:114425677	2,71172E-05	16.42	UncertainSignificance	VOUS
A026	M	MAP7D3	//	HEM	c.115G>A	p.Val39Met	X:135328456	0,000590842	15.76	UncertainSignificance	VOUS
A026	M	VSIG4	2	HEM	c.644G>A	p.Gly215Asp	X:65252360	1,12194E-05	18.44	UncertainSignificance	VOUS
A027	M	ZNF630	//	HEM	c.856A>G	p.Lys286Glu	X:47918603	0	22.1	UncertainSignificance	VOUS
A027	M	ICE1	//	COMP HET	c.791C>T	p.Thr264Ile	5:5457544	4,01916E-06	22.2	UncertainSignificance	VOUS
A027	M	ICE1	//	COMP HET	c.2170G>A	p.Glu724Lys	5:5461617	0	19.29	UncertainSignificance	VOUS
A027	M	COL4A6	//	HEM	c.998T>A	p.Ile333Asn	X:107437768	0,000264901	21.9	UncertainSignificance	VOUS
A027	M	DCAF8L1	//	HEM	c.370G>A	p.Gly124Ser	X:27999082	0,000529801	0.007	UncertainSignificance	LB
A027	M	LOC101059915	//	HEM	c.739C>T	p.Arg247Cys	X:70888392	0,000529801	0.665	UncertainSignificance	LB
A027	M	HDX	//	HEM	c.269G>A	p.Arg90Gln	X:83724462	0	10.13	UncertainSignificance	LB
A028	M	MAP3K15	//	HEM	c.2713G>A	p.Glu905Lys	X:19392655	1,68012E-05	18.30	UncertainSignificance	VOUS
A029	F	PRDM15	//	COMP HET	c.1156C>A	p.Leu386Ile	21:43279213	0	12.58	UncertainSignificance	LB
A029	F	PRDM15	//	COMP HET	c.295G>T	p.Gly99Cys	21:43298922	0,000510269	13.99	UncertainSignificance	LB
A029	F	AHNAK2	//	COMP HET	c.14018A>T	p.His4673Leu	14:105407770	0,002304147	0.13	UncertainSignificance	LB
A029	F	AHNAK2	//	COMP HET	c.386C>G	p.Thr129Arg	14:105421900	0,001181195	22.9	UncertainSignificance	VOUS
A029	F	REEP4	//	COMP HET	c.345C>G	p.Tyr115*	8:21997001	0	36.0	UncertainSignificance	VOUS
A029	F	REEP4	//	COMP HET	c.116T>C	p.Met39Thr	8:21997751	0,000116279	26.0	UncertainSignificance	VOUS
A033	M	MECP2	1	HEM	c.1160C>T	p.Pro387Leu	X:153296119	0,000264901	22.6	LikelyBenign	VOUS
A033	M	EIF4ENIF1	//	COMP HET	c.97G>A	p.Glu33Lys	22:31867903	0,0045	32	UncertainSignificance	VOUS
A033	M	EIF4ENIF1	//	COMP HET	c.865C>G	p.Gln289Glu	22:31854560	0,0006	25.7	UncertainSignificance	VOUS
A033	M	PCDH15	2	COMP HET	c.3502-8C>T		10:55626625	0,006162791	0.206	UncertainSignificance	VOUS
A033	M	PCDH15	2	COMP HET	c.2525A>G	p.Glu842Gly	10:55782653	3,99527E-06	34	UncertainSignificance	VOUS
A034	M	CDKL5	1	HEM	c.2779T>A	p.Ser927Thr	X:18646773	4,76974E-05	18	UncertainSignificance	VOUS
A034	M	EP400	2	COMP HET	c.8419C>G	p.Pro2807Ala	12:132549297	4,02411E-06	16,94	UncertainSignificance	VOUS
A034	M	EP400	2	COMP HET	c.2057C>T	p.Ser686Phe	12:132471186	0,000199681	20,7	UncertainSignificance	LP
A035	M	SOX3	//	HEM	c.310G>A	p.Gly104Arg	X:139586916	0,00014	24.8	UncertainSignificance	VOUS
A035	M	UNC80	2	COMP HET	c.92+7G>A		2:210636895	0,005643192	16.33	UncertainSignificance	VOUS
A035	M	UNC80	2	COMP HET	c.8011C>A	p.Leu2671Met	2:210835634	0	24.7	UncertainSignificance	VOUS
A037	M	WDFY4	2	COMP HET	c.2689A>C	p.Ser897Arg	10:49982638	0,000191083	0,043	UncertainSignificance	VOUS
A037	M	WDFY4	2	COMP HET	c.5114A>G	p.Asn1705Ser	10:50019653	0,000314268	0,275	UncertainSignificance	VOUS
A037	M	AKAP9	2	HETdn	c.1903G>A	p.Glu635Lys	7:91631134	0	31	UncertainSignificance	LP
A038	F	EPPK1	2	COMP HET	c.6193T>A	p.Tyr2065Asn	8:144941229	0,004125413	22.5	UncertainSignificance	VOUS
A038	F	EPPK1	2	COMP HET	c.5101G>A	p.Val1701Met	8:144942321	0,012664277	24.8	UncertainSignificance	VOUS
A041	M	CDKL5	1	HEM	c.826-6C>T		X:18616576	4,91951E-05	13.9	UncertainSignificance	VOUS
A041	M	APBA2	2	HETdn	c.916A>G	p.Thr306Ala	15:29347003	0	21.6	UncertainSignificance	LP
A041	M	TAF1C	3	COMP HET	c.1903G>A	p.Gly635Ser	16:84213254	0,008618682	14.99	UncertainSignificance	VOUS
A041	M	TAF1C	3	COMP HET	c.911C>T	p.Ser304Phe	16:84215475	0,00372093	13,38	UncertainSignificance	VOUS
A044	M	NEXMIF	1	HEM	c.2472G>T	p.Leu824Phe	X:73961920	0	22.8	UncertainSignificance	LP
A044	M	HIVEP3	2	COMP HET	c.3235C>A	p.Pro1079Thr	1:42047234	0,000453926	0,078	UncertainSignificance	VOUS
A044	M	HIVEP3	2	COMP HET	c.1600C>G	p.Pro534Ala	1:42048869	0,000814143	19,39	UncertainSignificance	VOUS
A044	M	FAT1	2	COMP HET	c.11497A>G	p.Thr3833Ala	4:187522566	0,000803484	24.3	UncertainSignificance	VOUS
A044	M	FAT1	2	COMP HET	c.3252A>G	p.Ile1084Met	4:187627730	0,002123142	23.0	UncertainSignificance	VOUS
A045	M	CNGB3	2	COMP HET	c.1578+1G>A		8:87638210	0,00023	29.7	UncertainSignificance	VOUS
A045	M	CNGB3	2	COMP HET	c.319G>A	p.Gly107Arg	8:87738778	0,00093	10.15	UncertainSignificance	VOUS
A046	M	SCN9A	2	COMP HET	c.3799C>G	p.Leu1267Val	2:167089942	0,002290813	24.5	UncertainSignificance	VOUS
A046	M	SCN9A	2	COMP HET	c.2987G>A	p.Arg996His	2:167129240	0,002213614	5.633	UncertainSignificance	VOUS
A048	M	CHD7	1	HOM	c.1018A>G	p.Met340Val	8:61655009	0,007448342	16.20	LikelyBenign	VOUS
A049	F	PTK7	1	HOM	c.19G>A	p.Gly7Arg	6:43044640	0,009279337	14.55	UncertainSignificance	VOUS
A049	F	CNOT3	1	HOM	c.845C>T	p.Ser282Phe	19:54650344	6,37105E-05	23.4	LikelyBenign	VOUS
A049	F	AGAP2	2	COMP HET	c.329T>C	p.Met110Thr	12:58128166	0,00255814	25.1	UncertainSignificance	VOUS
A049	F	AGAP2	2	COMP HET	c.966G>C	p.Glu322Asp	12:58131064	8,11879E-06	21.4	UncertainSignificance	VOUS

A050	F	TBC1D24	//	COMP HET	c.1349C>T	p.Pro450Leu	16:2550333	0,00023855	8.261	UncertainSignificance	LB
A050	F	TBC1D24	//	COMP HET	c.1393G>A	p.Ala465Thr	16:2550377	0,000209431	0.143	UncertainSignificance	LB
A051	F	LRP1	2	COMP HET	c.5795-8A>G		12:57577550	0	5.981	UncertainSignificance	VOUS
A051	F	LRP1	2	COMP HET	c.8304C>A	p.His2768Gln	12:57588880	0	16.97	UncertainSignificance	VOUS
A051	F	ANKMY1	//	COMP HET	c.1879-1G>A		2:241451419	0	22.5	Pathogenic	LP
A051	F	ANKMY1	//	COMP HET	c.638A>G	p.Asn213Ser	2:241468502	0,00047318	16.21	UncertainSignificance	VOUS
A051	F	SIDT2	//	COMP HET	c.1601A>G	p.Asn534Ser	11:117060989	0,000284154	23.9	UncertainSignificance	VOUS
A051	F	SIDT2	//	COMP HET	c.2128C>T	p.Arg710Cys	11:117063891	0,002793296	33	UncertainSignificance	VOUS
A051	F	SH3TC1	//	COMP HET	c.284G>T	p.Ser95Ile	4:8214464	1,21104E-05	19.47	UncertainSignificance	VOUS
A051	F	SH3TC1	//	COMP HET	c.1684C>T	p.Leu562Phe	4:8229105	0,001943418	23.9	UncertainSignificance	VOUS
A051	F	TBC1D9B	//	COMP HET	c.2455G>A	p.Glu819Lys	5:179298491	0,00372093	32	UncertainSignificance	VOUS
A051	F	TBC1D9B	//	COMP HET	c.686C>G	p.Ser229Cys	5:179320359	0,006395349	26.6	UncertainSignificance	VOUS
A051	F	ZSCAN21	//	COMP HET	c.133C>A	p.Arg45Ser	7:99654762	0,00019486	24.5	UncertainSignificance	VOUS
A051	F	ZSCAN21	//	COMP HET	c.796G>A	p.Val266Ile	7:99661614	0,000453926	13.48	UncertainSignificance	LB
A052	F	ANK3	1	COMP HET	c.2380G>T	p.Asp794Tyr	10:61819543	0,006976744	20.1	UncertainSignificance	VOUS
A052	F	ANK3	1	COMP HET	c.10055A>G	p.Glu3352Gly	10:61830584	0,005813953	21.0	LikelyBenign	VOUS
A052	F	IQGAP3	2	COMP HET	c.3277C>T	p.Arg1093Cys	1:156508605	3,18492E-05	32	UncertainSignificance	VOUS
A052	F	IQGAP3	2	COMP HET	c.3056+1G>A		1:156509165	0	25.8	Pathogenic	LP
A052	F	PDE4DIP	//	COMP HET	c.6905A>T	p.Asn2302Ile	1:144854565	0,001046512	17.31	UncertainSignificance	VOUS
A052	F	PDE4DIP	//	COMP HET	c.907G>C	p.Val303Leu	1:144922261	0	24.3	UncertainSignificance	VOUS
A053	M	PHF8	S	HEM	c.1379T>A	p.Ile460Asn	X:54020281	1,09716E-05	21.9	UncertainSignificance	LP
A053	M	FHDC1	//	COMP HET	c.1453C>T	p.Arg485Cys	4:153895896	0,000232558	16.23	UncertainSignificance	VOUS
A053	M	FHDC1	//	COMP HET	c.2854G>A	p.Ala952Thr	4:153897297	1,22245E-05	0.135	UncertainSignificance	VOUS
A053	M	PNPLA7	2	COMP HET	c.1906G>A	p.Gly636Arg	9:140391671	3,18492E-05	24.7	UncertainSignificance	VOUS
A053	M	PNPLA7	2	COMP HET	c.1421G>A	p.Arg474Gln	9:140396157	0,000798722	23.9	UncertainSignificance	VOUS
A053	M	RHOXF1	2	HEM	c.190G>A	p.Gly64Ser	X:119249583	1,17861E-05	14.60	UncertainSignificance	VOUS
A054	M	SYN1	1	HEM	c.1779A>C	p.Lys593Asn	X:47433604	0	18.03	UncertainSignificance	VOUS
A054	M	ADCY9	//	COMP HET	c.2828+4C>T		16:4027479	3,61485E-05	0.025	UncertainSignificance	LB
A054	M	ADCY9	//	COMP HET	c.2827A>G	p.Ser943Gly	16:4027484	0	24.5	UncertainSignificance	VOUS
A054	M	ESPL1	//	COMP HET	c.130G>T	p.Ala44Ser	12:53662856	0	17.57	UncertainSignificance	VOUS
A054	M	ESPL1	//	COMP HET	c.1846G>A	p.Glu616Lys	12:53670549	0,000127405	28.7	UncertainSignificance	VOUS
A054	M	FAM47A	2	HEM	c.1499G>A	p.Arg500Gln	X:34148897	0,000148876	14.68	UncertainSignificance	VOUS
A055	M	SYNE1	2S	COMP HET	c.6521C>T	p.Thr2174Ile	6:152730222	0,001264816	19.00	UncertainSignificance	VOUS
A055	M	SYNE1	2S	COMP HET	c.4822G>A	p.Ala1608Thr	6:152749494	0,001627907	0.813	UncertainSignificance	VOUS
A056	M	MCPH1	2	HETdn	c.1477C>T	p.Arg493Cys	8:6302720	3,18451E-05	0.001	UncertainSignificance	VOUS
A057	M	MAGEE2	//	HEM	c.181G>A	p.Ala61Thr	X:75004706	0	8.479	UncertainSignificance	VOUS
A057	M	KDM6B	1	COMP HET	c.1925C>T	p.Pro642Leu	17:7751531	0,003947052	22.1	UncertainSignificance	VOUS
A057	M	KDM6B	1	COMP HET	c.2638C>G	p.Arg880Gly	17:7752244	0,000480307	23.5	UncertainSignificance	VOUS
A058	M	MAGEE2	//	HEM	c.181G>A	p.Ala61Thr	X:75004706	0	8.479	UncertainSignificance	VOUS
A059	M	MAGEE2	//	HEM	c.181G>A	p.Ala61Thr	X:75004706	0	8.479	UncertainSignificance	VOUS
A060	M	MAP7D2	//	HEM	c.619A>G	p.Met207Val	X:20069041	6,25006E-05	8.170	UncertainSignificance	LB
A060	M	BIRC6	2	HETdn	c.1687C>A	p.Pro563Thr	2:32640046	0	23.3	UncertainSignificance	VOUS
A060	M	DDX53	2	HEM	c.1678C>T	p.Arg560Trp	X:23019852	5,5001E-06	23.3	UncertainSignificance	VOUS
A062	M	RNF19A	//	COMP HET	c.1147A>G	p.Ile383Val	8:101281057	0,000453926	18.61	UncertainSignificance	VOUS
A062	M	RNF19A	//	COMP HET	c.1202G>A	p.Arg401His	8:101277003	0,001395349	26.2	UncertainSignificance	VOUS
A064	F	RGSL1	//	HETdn	c.2705A>T	p.Gln902Leu	1:182517487	0	9.319	Likelypathogenic	VOUS
A066	F	CGNL1	2	COMP HET	c.1702A>G	p.Thr568Ala	15:57734575	0,00729927	2.937	LikelyBenign	LB
A066	F	CGNL1	2	COMP HET	c.3049A>G	p.Met1017Val	15:57820861	0,004310345	14.17	LikelyBenign	LB
A066	F	LTK	//	COMP HET	c.2416G>A /	p.Gly806Arg	15:41796373	0,00012	14.82	UncertainSignificance	VOUS
A066	F	LTK	//	COMP HET	c.1237G>A /	p.Val413Ile	15:41800279	0,000064	14.39	UncertainSignificance	VOUS
A067	M	GPHN	2	HOM	c.26C>G	p.Thr9Ser	14:66975271	0,001976744	21.7	LikelyBenign	VOUS
A067	M	SYNE1	2S	HETdn	c.18481G>A	p.Glu6161Lys	6:152599316	0,00	23.9	LikelyBenign	VOUS
A068	F	EPPK1	2	COMP HET	c.5893C>T	p.Arg1965Trp	8:144941529	0,000597086	22.9	UncertainSignificance	VOUS

A068	F	EPPK1	2	COMP HET	c.5101G>A	p.Val1701Met	8:144942321	0,012664277	24.8	UncertainSignificance	VOUS
A068	F	CASZ1	1	COMP HET	c.845C>T	p.Thr282Met	1:10720254	0,008025122	22.4	LikelyBenign	VOUS
A068	F	CASZ1	1	COMP HET	c.794T>C	p.Val265Ala	1:10720305	0,009767442	1.032	LikelyBenign	LB
A069	M	MCF2	//	HEM	c.911T>C	p.Leu304Pro	X:138701822	0	23.9	UncertainSignificance	VOUS
A069	M	CLDN34	//	HEM	c.604C>A	p.Pro202Thr	X:9936001	0	9.123	UncertainSignificance	VOUS
A070	M	LRCH2	//	HEM	c.1415A>G	p.Asp472Gly	X:114398287	0	24.7	UncertainSignificance	VOUS
A070	M	ABCA13	2	COMP HET	c.9236A>G	p.Lys3079Arg	7:48337999	8,07735E-06	0.036	LikelyBenign	LB
A070	M	ABCA13	2	COMP HET	c.14185C>T	p.Arg4729Cys	7:48563977	0,000611247	7.439	LikelyBenign	LB
A070	M	TAF6	2	HOM	c.2122G>A	p.Gly708Ser	7:99704892	0,005138986	13.50	LikelyBenign	LB
A070	M	SLITRK4	//	HEM	c.799G>A	p.Gly267Ser	X:142718126	0,000264901	22.1	LikelyBenign	VOUS
A070	M	SUPV3L1	//	HOM	c.505A>G	p.Ile169Val	10:70947445	3,19244E-05	20.5	UncertainSignificance	VOUS
A070	M	DST	2	COMP HET	c.11924A>G	p.Asp3975Gly	6:56382072	0	32	UncertainSignificance	VOUS
A070	M	DST	2	COMP HET	c.7534G>C	p.Gly2512Arg	6:56480731	3,9807E-06	15.90	UncertainSignificance	VOUS
A071	M	AGBL4	2	COMP HET	c.101G>T	p.Gly34Val	1:50317124	0,007225434	24.6	UncertainSignificance	VOUS
A071	M	AGBL4	2	COMP HET	c.-41C>T		1:50489509	0,003612717	11.71	UncertainSignificance	VOUS
A071	M	STXBP5	2	COMP HET	c.787A>G	p.Ile263Val	6:147599289	0,002269632	16.80	UncertainSignificance	VOUS
A071	M	STXBP5	2	COMP HET	c.1505A>G	p.Tyr502Cys	6:147636753	0,005813953	21.3	LikelyBenign	VOUS
A072	M	AGBL4	2	COMP HET	c.101G>T	p.Gly34Val	1:50317124	0,007225434	24.6	UncertainSignificance	VOUS
A072	M	AGBL4	2	COMP HET	c.-41C>T		1:50489509	0,003612717	11.71	UncertainSignificance	VOUS
A073	M	AGBL4	2	COMP HET	c.101G>T	p.Gly34Val	1:50317124	0,007225434	24.6	UncertainSignificance	VOUS
A073	M	AGBL4	2	COMP HET	c.-41C>T		1:50489509	0,003612717	11.71	UncertainSignificance	VOUS
A074	M	MTUS1	//	COMP HET	c.1655T>C	p.Val552Ala	8:17611662	0,000116345	7.077	UncertainSignificance	LB
A074	M	MTUS1	//	COMP HET	c.312G>T	p.Gln104His	8:17613005	0,000727096	14.94	LikelyBenign	LB
A074	M	KIF15	//	COMP HET	c.1502G>T	p.Arg501Leu	3:44843459	0,003439271	27.1	LikelyBenign	VOUS
A074	M	KIF15	//	COMP HET	c.4020T>G	p.Asn1340Lys	3:44893747	3,97985E-06	23.3	UncertainSignificance	VOUS
A075	F	EP400	2	HOM	c.8181_8182insGCAG	p.Gln2728fs*255	12:132547093	0	26.9	LikelyPathogenic	LP
A075	F	KIF13B	2	COMP HET	c.3821A>G	p.Asn1274Ser	8:28974364	0,0261916	9.538	LikelyBenign	LB
A075	F	KIF13B	2	COMP HET	c.2411A>G	p.Asn804Ser	8:28998058	0,00103146	24.9	UncertainSignificance	VOUS
A075	F	YIF1A	//	HETdn	c.19T>C	p.Tyr7His	11:66056438	6,41067E-05	25.0	UncertainSignificance	VOUS
A076	M	FTSJ1	//	HEM	c.111A>C	p.Gln37His	X:48336546	5,75E-06	0.845	LikelyBenign	LB
A076	M	STAB1	//	HETdn	c.3357_3358insC	p.Arg1122fs*36	3:52547907	0,00140647	32	UncertainSignificance	VOUS
A076	M	DGKB	//	HETdn	c.2207C>T	p.Ala736Val	7:14217695	0	32	LikelyPathogenic	LP
A076	M	ACTR5	//	COMP HET	c.911G>A	p.Arg304Gln	20:37383735	1,1957E-05	34.0	UncertainSignificance	VOUS
A076	M	ACTR5	//	COMP HET	c.1697A>G	p.Lys566Arg	20:37400332	1,19301E-05	22.3	UncertainSignificance	VOUS
A076	M	WAS	//	HEM	c.724A>T	p.Ser242Cys	X:48545334	8,46131E-05	24.7	UncertainSignificance	VOUS
A077	M	IL1RAPL1	2	HEM	c.261G>C	p.Glu87Asp	X:29301233	1,09092E-05	22.9	LikelyBenign	VOUS
A077	M	HECTD4	1	COMP HET	c.11749G>A	p.Gly3917Ser	12:112607364	0	23.2	UncertainSignificance	VOUS
A077	M	HECTD4	1	COMP HET	c.4112-5dupT		12:112681817	0,00259585	10.95	UncertainSignificance	VOUS
A077	M	ACTN4	3	COMP HET	c.1443-4G>A		19:39214250	0,000199681	7.064	UncertainSignificance	VOUS
A077	M	ACTN4	3	COMP HET	c.2680G>A	p.Gly894Ser	19:39220016	0,014	24.9	UncertainSignificance	VOUS
A078	M	POPDC2	//	HETdn	c.126G>C	p.Met42Ile	3:119379145	0	27.2	LikelyPathogenic	LP
A078	M	MSC	//	HETdn	c.100G>A	p.Val34Ile	8:72756314	0	15.69	LikelyPathogenic	VOUS
A078	M	MOCS2	//	HOM	c.14G>A	p.Cys5Tyr	5:52405546	3.12e-4	17.43	UncertainSignificance	LP
A079	M	SMARCA1	//	HEM	c.3079A>T	p.Asn1027Tyr	X:128582372	5,50E-06	24.6	UncertainSignificance	LP
A079	M	DDX20	//	COMP HET	c.25G>A	p.Gly9Arg	1:112298571	0	15.74	UncertainSignificance	VOUS
A079	M	DDX20	//	COMP HET	c.412C>T	p.Pro138Ser	1:112302037	1,27294E-05	26.5	UncertainSignificance	VOUS
A079	M	RHBDD3	//	COMP HET	c.968C>T	p.Ser323Phe	22:29656330	3,679E-05	32	UncertainSignificance	VOUS
A079	M	RHBDD3	//	COMP HET	c.512delG	p.Gly171fs*40	22:29659843	0,002240478	32	UncertainSignificance	VOUS
A079	M	BSN	//	COMP HET	c.4585A>G	p.Met1529Val	3:49691574	0,000116279	18.19	UncertainSignificance	VOUS
A079	M	BSN	//	COMP HET	c.9256C>T	p.Pro3086Ser	3:49698534	0	11.89	UncertainSignificance	LB
A079	M	LUZP4	//	HEM	c.21A>G	p.Ile7Met	X:114536613	1,16158E-05	0.372	UncertainSignificance	VOUS
A080	M	PNCK	//	HEM	c.1189G>C	p.Gly397Arg	X:152936004	0,000148721	24.0	LikelyBenign	VOUS
A080	M	SPAST	1	COMP HET	c.415+4A>G		2:32289319	0	15.95	UncertainSignificance	VOUS

A080	M	SPAST	1	COMP HET	c.870+8delG		2:32339901	3,9814E-06	1.412	UncertainSignificance	VOUS
A080	M	FAAP100	//	HETdn	c.1435C>T	p.Arg479Trp	17:79514673	0,000116279	29.6	LikelyBenign	VOUS
A080	M	ARHGEF11	//	COMP HET	c.1912G>C	p.Asp638His	1:156918184	3,97687E-06	29.2	UncertainSignificance	VOUS
A080	M	ARHGEF11	//	COMP HET	c.329A>G	p.Lys110Arg	1:156949023	0	25.5	UncertainSignificance	VOUS
A080	M	RABGAP1L	//	COMP HET	c.1220G>A	p.Arg407His	1:174247814	7,99329E-06	32	UncertainSignificance	VOUS
A080	M	RABGAP1L	//	COMP HET	c.878C>T	p.Ser293Leu	1:174957893	0	22.9	UncertainSignificance	VOUS
A080	M	C1QTNF7	//	HETdn	c.716C>T	p.Ser239Leu	4:15444248	1,98915E-05	29.9	UncertainSignificance	VOUS
A082	M	KLHDC7A	//	HETdn	c.650delG	p.Ser217fs*2	1:18808124	0	22.3	LikelyPathogenic	VOUS
A082	M	WDFY3	1	COMP HET	c.9726+3A>G		4:85605093	0	12,65	UncertainSignificance	VOUS
A082	M	WDFY3	1	COMP HET	c.5189-7C>G		4:85678321	0,000530815	11,43	UncertainSignificance	VOUS
A083	M	LOC101059915	//	HEM	c.604delG	p.Leu203fs*0	X:70888256	0,0010596	0.022	UncertainSignificance	LB
A083	M	TKTL1	//	HEM	c.950A>C	p.His317Pro	X:153549192	0	14.76	UncertainSignificance	VOUS
A084	M	VCX3B	//	HEM	c.188C>T	p.Thr63Met	X:8433871	0,000082	0.076	UncertainSignificance	LB
A084	M	ERAS	//	HEM	c.91C>T	p.Arg31Cys	X:48687624	6,94845E-05	12.29	LikelyBenign	LB
A084	M	CHIC1	//	HEM	c.611A>C	p.Asn204Thr	X:72899535	9,18063E-05	26.3	LikelyBenign	VOUS
A084	M	COL16A1	//	COMP HET	c.4027C>T	p.Pro1343Ser	1:32122663	0,000119076	24.9	UncertainSignificance	VOUS
A084	M	COL16A1	//	COMP HET	c.280C>T	p.Arg94Trp	1:32164194	0,000724638	24.1	LikelyBenign	VOUS
A084	M	PASK	3	COMP HET	c.1838A>G	p.Tyr613Cys	2:242066492	0,000232558	9.502	UncertainSignificance	VOUS
A084	M	PASK	3	COMP HET	c.1576G>A	p.Gly526Arg	2:242066754	0,000697674	0.011	UncertainSignificance	VOUS
A086	M	CETN2	//	HEM	c.2T>C	p.Met1?	X:151999253	3,16704E-05	22.5	UncertainSignificance	VOUS
A087	M	BCORL1	S	HEM	c.3371A>G	p.Glu1124Gly	X:129150119	5,51557E-06	19.40	LikelyBenign	VOUS
A087	M	NEXMIF	1	HEM	c.1777_1779delAAG	p.Lys593del	X:73962612	2,74832E-05	17.57	UncertainSignificance	VOUS
A088	M	KLHL34	//	HEM	c.1507G>A	p.Glu503Lys	X:21674400	3,74462E-05	18.80	LikelyBenign	LB
A088	M	TAF1	S	HEM	c.3005C>T	p.Pro1002Leu	X:70612738	4,59601E-05	22.6	LikelyBenign	VOUS
A088	M	CAMK2D	//	HETdn	c.493C>T	p.Gln165*	4:114458521	0	40	LikelyPathogenic	LP
A093	M	PLXNA3	2	HEM	c.524C>T	p.Ser175Leu	X:153689047	0,000264901	25.1	UncertainSignificance	VOUS
A093	M	SMYD1	//	COMP HET	c.17_18delTG	p.Met6fs*8	2:88367399	0	34	UncertainSignificance	LP
A093	M	SMYD1	//	COMP HET	c.1253A>G	p.Lys418Arg	2:88407997	0,000813953	22.0	UncertainSignificance	VOUS
A094	M	PDE4DIP	//	COMP HET	c.5341C>T	p.Leu1781Phe	1:144868098	0,000599042	14.48	UncertainSignificance	VOUS
A094	M	PDE4DIP	//	COMP HET	c.1546C>T	p.Leu516Phe	1:144921972	0,000116387	25.6	UncertainSignificance	VOUS
A095	M	TRIM35	//	COMP HET	c.1279C>T	p.Arg427Cys	8:27145270	0,000399361	31	UncertainSignificance	VOUS
A095	M	TRIM35	//	COMP HET	c.1007C>T	p.Pro336Leu	8:27145542	8,01533E-06	25.7	UncertainSignificance	VOUS
A095	M	LRBA	2	HETdn	c.3656C>G	p.Thr1219Ser	4:151773206	0	6.985	LikelyPathogenic	VOUS
A096	F	GALNT8	2	COMP HET	c.151A>G	p.Lys51Glu	12:4829994	3,98E-06	5.002	UncertainSignificance	VOUS
A096	F	GALNT8	2	COMP HET	c.1241A>G	p.His414Arg	12:4870191	0	14.77	UncertainSignificance	VOUS
A096	F	TAF6	2	HETdn	c.1442C>T	p.Thr481Ile	7:99706006	0	24.8	LikelyPathogenic	LP
A097	M	RNF220	//	HETdn	c.261delT	p.Phe88fs*16	1:44878029	0	29.7	LikelyPathogenic	LP
A099	M	PLXNA3	2	HEM	c.859G>A	p.Val287Met	X:153689703	0,000148633	20.5	LikelyBenign	VOUS
A100	M	SAGE1	//	HEM	c.2306G>C	p.Ser769Thr	X:134993897	0	16.48	UncertainSignificance	VOUS
A100	M	RNF25	2	HOM	c.620A>C	p.Tyr207Ser	2:219529924	0,005232558	25.5	LikelyBenign	VOUS
A100	M	DOCK5	//	HETdn	c.778A>T	p.Ile260Phe	8:25158105	0	23.9	LikelyPathogenic	LP
A100	M	SPPL2C	//	COMP HET	c.511G>A	p.Val171Ile	17:43922783	3,18613E-05	1.584	UncertainSignificance	LB
A100	M	SPPL2C	//	COMP HET	c.1716_1719delAGAC	p.Asp573fs*51	17:43923987	0,001172608	24.3	UncertainSignificance	VOUS
A100	M	CSMD1	2	COMP HET	c.1223-5T>C		8:3432593	4,04E-06	10.87	UncertainSignificance	VOUS
A100	M	CSMD1	2	COMP HET	c.1184C>T	p.Thr395Met	8:3443696	0,000258264	15.38	UncertainSignificance	VOUS
A101	M	ARHGAP6	//	HEM	c.1524A>G	p.Ile508Met	X:11196325	0	21.2	UncertainSignificance	VOUS
A102	M	IL1RAPL1	2	HEM	c.11C>T	p.Pro4Leu	X:28807471	0,000136475	21.5	LikelyBenign	VOUS
A102	M	COPS5	//	HETdn	c.643G>A	p.Gly215Ser	8:67968770	0	32	LikelyPathogenic	LP
A102	M	CES3	//	COMP HET	c.422G>A	p.Arg141Lys	16:66997528	3,18613E-05	6.518	UncertainSignificance	LB
A102	M	CES3	//	COMP HET	c.506G>A	p.Gly169Glu	16:66997784	0,000909091	12.04	UncertainSignificance	LB
A103	M	EP400	2	COMP HET	c.3469G>A	p.Val1157Ile	12:132497581	0,004534884	12.97	LikelyBenign	LB
A103	M	EP400	2	COMP HET	c.8223_8224insCAGCAA	p.Gln2741_Gln2742dup	12:132547135	0,000321048	14.27	UncertainSignificance	VOUS
A103	M	LILRB2	2	COMP HET	c.940G>A	p.Asp314Asn	19:54782682	0,005368441	23.2	LikelyBenign	VOUS

A103	M	LILRB2	2	COMP HET	c.67A>G	p.Thr23Ala	19:54784122	0,0227636	4.512	UncertainSignificance	VOUS
A104	M	GPC4	2	HEM	c.1388G>A	p.Arg463His	X:132437274	9,11328E-05	25.1	LikelyBenign	VOUS
A105	M	SYN1	1	HEM	c.1970A>C	p.His657Pro	X:47433413	0	23.4	UncertainSignificance	LP
A107	M	CACNA1F	2	HEM	c.795G>C	p.Lys265Asn	X:49086704	0,000148633	24.3	LikelyBenign	VOUS
A108	M	CTTNBP2	2	HETdn	c.1725_1726delAG	p.Ala577fs*2	7:117431523	0	34	LikelyPathogenic	P
A108	M	CASR	//	HETdn	c.102_103insG	p.Leu37fs*10	3:121973138	0	32	UncertainSignificance	LP
A109	M	SYNE1	2S	COMP HET	c.25469T>C	p.Leu8490Pro	6:152457799	1,19405E-05	27.4	UncertainSignificance	VOUS
A109	M	SYNE1	2S	COMP HET	c.17567C>T	p.Pro5856Leu	6:152615165	0,000226963	17.07	UncertainSignificance	VOUS
A109	M	SYNE1	2S	COMP HET	c.12261G>C	p.Arg4087Ser	6:152658030	3,97769E-06	23.6	UncertainSignificance	VOUS
A109	M	SYNE1	2S	COMP HET	c.10043T>C	p.Val3348Ala	6:152686105	0,028597367	22.5	LikelyBenign	VOUS
A109	M	EP400	2	COMP HET	c.5324G>T	p.Ser1775Ile	12:132512776	0,007943713	8.32	LikelyBenign	LB
A109	M	EP400	2	COMP HET	c.8399C>T	p.Ala2800Val	12:132549277	0,000199681	23.4	UncertainSignificance	VOUS
A110	M	GPR50	//	HEM	c.1142G>A	p.Arg381His	X:150349197	5,49949E-06	2.745	LikelyBenign	LB
A110	M	CASZ1	1	HOM	c.794T>C	p.Val265Ala	1:10720305	0,009767442	1.032	LikelyBenign	LB
A110	M	RAI1	1	COMP HET	c.131G>A	p.Arg44Gln	17:17696393	0,000599042	24.6	UncertainSignificance	LP
A110	M	RAI1	1	COMP HET	c.2939C>A	p.Ala980Asp	17:17699201	0,000116306	23.2	UncertainSignificance	LP
A110	M	BAHCC1	//	COMP HET	c.379G>A	p.Val127Met	17:79408925	0,000606649	9.539	UncertainSignificance	LB
A110	M	BAHCC1	//	COMP HET	c.6070G>A	p.Gly2024Ser	17:79427678	0,000399361	1.298	UncertainSignificance	LB
A111	M	ABCA13	2	COMP HET	c.10202A>T	p.Tyr3401Phe	7:48378050	0	13.71	LikelyBenign	LB
A111	M	ABCA13	2	COMP HET	c.14200A>T	p.Ile4734Phe	7:48563992	6,05175E-05	16.86	LikelyBenign	VOUS
A111	M	LLGL1	//	COMP HET	c.382G>A	p.Asp128Asn	17:18136106	0	24.3	UncertainSignificance	VOUS
A111	M	LLGL1	//	COMP HET	c.329G>A	p.Cys110Tyr	17:18136053	7,95E-06	17.45	UncertainSignificance	VOUS
A112	M	RPA4	//	HEM	c.620G>A	p.Arg207His	X:96139929	0	11.03	UncertainSignificance	VOUS
A112	M	FOCAD	//	COMP HET	c.3337A>T	p.Met1113Leu	9:20933032	0	18.20	UncertainSignificance	VOUS
A112	M	FOCAD	//	COMP HET	c.4440A>C	p.Glu1480Asp	9:20981487	0	15.21	UncertainSignificance	VOUS
A112	M	ANOS1	//	HEM	c.1138T>C	p.Trp380Arg	X:8536342	0	24.3	UncertainSignificance	LP
A112	M	SYTL4	//	HEM	c.428A>G	p.Lys143Arg	X:99956224	0	10.52	UncertainSignificance	VOUS
A114	M	CDH22	2	HETdn	c.673G>A	p.Val225Ile	20:44845630	0,000199681	14.58	LikelyBenign	VOUS
A115	M	ABCB7	//	HEM	c.10C>T	p.Leu4Phe	X:74376098	0	23.6	UncertainSignificance	VOUS
A116	M	CTPS2	//	HEM	c.1442G>A	p.Arg481Gln	X:16638407	1,10434E-05	26.5	UncertainSignificance	VOUS
A116	M	ANK2	1	COMP HET	c.877G>A	p.Asp293Asn	4:114163351	0,000115342	27.6	UncertainSignificance	VOUS
A116	M	ANK2	1	COMP HET	c.8979A>G	p.Ile2993Met	4:114278753	0,002950522	0.170	UncertainSignificance	VOUS
A117	M	SPTBN5	//	COMP HET	c.8836G>A	p.Glu2946Lys	15:42148769	1,85242E-05	22.1	UncertainSignificance	VOUS
A117	M	SPTBN5	//	COMP HET	c.1829T>C	p.Leu610Pro	15:42175257	4,98927E-06	24.4	UncertainSignificance	VOUS
A118	M	GEMIN4	//	COMP HET	c.2845C>T	p.Arg949Cys	17:648438	0,00129199	22.6	UncertainSignificance	VOUS
A118	M	GEMIN4	//	COMP HET	c.812C>T	p.Thr271Met	17:650471	0,00023116	23.6	UncertainSignificance	VOUS
A118	M	GABBR1	//	HETdn	c.1939dupC	p.Leu647fs*28	6:29576430	0	34	LikelyPathogenic	LP
A120	M	CXorf58	//	HEM	c.116+1G>A		X:23928536	9,11037E-05	23	UncertainSignificance	VOUS
A120	M	ARID1B	1	COMP HET	c.364_366delCAG	p.Gln122del	6:157099426	0,033088235	18.27	LikelyBenign	VOUS
A120	M	ARID1B	1	COMP HET	c.2986+6C>T		6:157488325	0,005470205	3.74	UncertainSignificance	VOUS
A120	M	LRP2	2	HETdn	c.6954C>A	p.Asp2318Glu	2:170063276	0	23.2	LikelyPathogenic	LP
A120	M	DST	2	COMP HET	c.12220C>T	p.Arg4074Cys	6:56362659	8,19101E-06	32.0	UncertainSignificance	VOUS
A120	M	DST	2	COMP HET	c.772C>T	p.Pro258Ser	6:56515753	4,02117E-06	21.9	UncertainSignificance	VOUS
A121	F	SCN2A	1	HETdn	c.5148G>T	p.Trp1716Cys	2:166245464	0	33	LikelyPathogenic	P
A122	M	NSDHL	//	HEM	c.263G>A	p.Arg88Gln	X:152018963	2,1823E-05	11.90	UncertainSignificance	VOUS
A122	M	MXRA5	//	HEM	c.6328C>A	p.Arg2110Ser	X:3235394	0	24.8	UncertainSignificance	VOUS
A123	**F**	DCAF1	//	HETdn	c.2270C>T	p.Ala757Val	3:51457995	0	25	UncertainSignificance	VOUS
A124	M	TSC2	1	COMP HET	c.1318G>A	p.Gly440Ser	16:2112558	0,001627907	25.1	UncertainSignificance	VOUS
A124	M	TSC2	1	COMP HET	c.5378G>A	p.Arg1793Gln	16:2138565	0,000145605	26.4	UncertainSignificance	VOUS
A124	M	HECW2	2	HETdn	c.3493C>T	p.Arg1165Cys	2:197118680	3,1837E-05	32	LikelyPathogenic	P
A125	M	GABRE	//	HEM	c.983G>A	p.Arg328His	X:151123994	1,09154E-05	23.6	UncertainSignificance	LP
A125	M	KDM6B	1	COMP HET	c.2477C>G	p.Ser826Cys	17:7752083	0,000116306	23.7	LikelyBenign	VOUS
A125	M	KDM6B	1	COMP HET	c.3431G>A	p.Arg1144His	17:7753037	2,03618E-05	29.0	LikelyBenign	VOUS

A125	M	ZNF462	1	COMP HET	c.3500C>T	p.Pro1167Leu	9:109689693	3,99288E-06	23	UncertainSignificance	LP
A125	M	ZNF462	1	COMP HET	c.5077A>G	p.Lys1693Glu	9:109691270	0	25.5	UncertainSignificance	LP
A128	M	SYNE1	2S	COMP HET	c.15238G>A	p.Val5080Met	6:152646425	0,000998393	15.88	LikelyBenign	LB
A128	M	SYNE1	2S	COMP HET	c.4631G>A	p.Arg1544Gln	6:152751696	0,00459265	18.42	LikelyBenign	LB
A129	M	MAOB	2	HEM	c.1558G>A	p.Val520Ile	X:43626718	0	0.003	UncertainSignificance	VOUS
A129	M	SYTL4	//	HEM	c.540-1G>A		X:99946207	0,000149316	29.3	UncertainSignificance	VOUS
A130	M	PLEKHA2	//	HETdn	c.689_690insC	p.Lys231fs*2	8:38810209	0	35	UncertainSignificance	VOUS
A130	M	PAK3	//	HEM	c.525_526insGAAGAG	p.Glu176_Glu177insGluGlu	X:110406199	0	21	UncertainSignificance	LP
A130	M	RANBP17	2	COMP HET	c.692T>C	p.Ile231Thr	5:170338070	0,00339457	24.5	LikelyBenign	VOUS
A130	M	RANBP17	2	COMP HET	c.3244T>C	p.Cys1082Arg	5:170725839	3,18431E-05	11.58	UncertainSignificance	VOUS
A130	M	RBL1	//	HETdn	c.2011A>G	p.Met671Val	20:35663804	0	13.17	UncertainSignificance	VOUS
A131	M	DDX53	2	HEM	c.543A>C	p.Glu181Asp	X:23018717	1,09765E-05	23.2	LikelyBenign	VOUS
A131	M	AR	2	HEM	c.170_171insGCA	p.Leu57_Gln58insGln	X:66765158	0	13.6	LikelyBenign	VOUS
A132	M	KCND1	//	HEM	c.1368+1G>A		X:48823167	0	27.9	UncertainSignificance	LP
