# Supplementary material for: Unveiling genetic insights: Array-CGH and WES discoveries in a cohort of 122 children with essential autism spectrum disorder
Source: BMC Genomics. 2024 Dec 10;25:1186. doi: 10.1186/s12864-024-11077-5 (PMC11629504; doi:10.1186/s12864-024-11077-5)
Supplement: Supplementary file 2 — Supplementary Material 2 [file 12864_2024_11077_MOESM2_ESM.pdf]

Supplementary Table 2. Copy number variants identified by array comparative genomic hybridization.

CASE	SEX	CNV - ISCN 2020	SIZE	CNV CLASSIFICATION	GENES	Genes of Interest	SFARI score	TYPE OF GENE REARRANGEMENT
A003	M	13q14.13q14.2(47240527_48096722)x1 pat	856.2 kb	VOUS	*LRCH1*		//	INT START
A003					*ESD*		//	DELETED
A003					*HTR2A*	*HTR2A*	//	DELETED
A006	M	13q12.11(20146801_20270834)x3 pat	124.03 kb	VOUS	*MPHOSPH8*	*MPHOSPH8*	//	DUPLICATED
A006					*PSPC1*		//	INT END
A006		13q12.11(20411945_20559030)x3 pat	147.09 kb	VOUS	*ZMYM5*	*ZMYM5*	//	INT START
A006					*ZMYM2*	*ZMYM2*	S	INT END
A006		17q21.31 (41409849_41597925)x3 mat	188.08 kb	VOUS	*DHX8*	*DHX8*	//	INT END
A013	M	7q11.22(69168540_69198438)x1 mat	29.9 kb	LP	*AUTS2*	*AUTS2*	1	INTRAGENIC
A016	M	16p13.11 (14968878_16311041)X1 dn	1.34 Mb	LP	*NOMO1*		//	INT START
A016					*NPIPA1*		//	DELETED
A016					*PDXDC1*		//	DELETED
A016					*NTAN1*		//	DELETED
A016					*RRN3*		//	DELETED
A016					*MPV17L*		//	DELETED
A016					*MARF1*		//	DELETED
A016					*NDE1*	*NDE1*	//	DELETED
A016					*MYH11*		//	DELETED
A016					*FOPNL*		//	DELETED
A016					*ABCC1*		//	DELETED
A016					*ABCC6*		//	INT END
A017	M	16q21(60064996_63555913)x1 mat	3.49 Mb	LP	*CDH8*	*CDH8*	2	DELETED
A020	M	3q24(147111840_147118109)x3 dn	8.91 kb	VOUS	*ZIC1*	*ZIC1*	//	INTRAGENIC
A020					*ZIC4*		//	INTRAGENIC
A021	F	15q13.1(29656854_29783953)x1 pat	127.1 kb	VOUS	*FAM189A1*	*FAM189A1*	//	INTRAGENIC
A022	M	Xp22.33(426261_679415)x2 mat	253.16 kb	LP	*SHOX*	*SHOX*	2	DUPLICATED
A025	M	16q23.3(82184809_83663330)x3 mat	1.48 Mb	VOUS	*CDH13*	*CDH13*	2	INT END
A025					*MPHOSPH6*		//	INT START
A029	F	7q21.11(81472446_81639364)x3 pat	166.92 kb	VOUS	*CACNA2D1*	*CACNA2D1*	2	INT END
A043	M	16q23.2(80751184_80928051)x1 mat	176.87 kb	VOUS	*CDYL2*	*CDYL2*	//	INT START
A045	M	Xq21.1(77081624_77139044)x2 mat	57.42 kb	VOUS	*MAGT1*	*MAGT1*	//	INT END
A050	F	1q21.1q21.2(146542653_147872312)x1 pat	1.33 Mb	LP	*PRKAB2*		//	DELETED
A050					*FMO5*		//	DELETED
A050					*BCL9*		//	DELETED
A050					*CHD1L*		//	DELETED
A050					*ACP6*		//	DELETED
A050					*GJA5*		//	DELETED
A050					*GJA8*		//	DELETED
A050					*GPR89B*		//	DELETED
A054	M	1q21.1q21.2(146499008_147860611)x3 pat	1.36 Mb	LP	*PRKAB2*		//	DUPLICATED
A054					*FMO5*		//	DUPLICATED
A054					*BCL9*		//	DUPLICATED
A054					*CHD1L*		//	DUPLICATED
A054					*ACP6*		//	DUPLICATED
A054					*GJA5*		//	DUPLICATED
A054					*GJA8*		//	DUPLICATED

A054					*GPR89B*		//	DUPLICATED
A060	M	6q22.31(124068036_124449980)x3 mat	382 Kb	VOUS	*NKAIN2*	*NKAIN2*	//	INT END
A060		11q25(133124721_134353783)x3 mat	1.23 Mb	VOUS	*OPCML*		//	INT START
A060					*SPATA19*		//	DUPLICATED
A060					*IGSF9B*		//	DUPLICATED
A060					*JAM3*		//	DUPLICATED
A060					*NCAPD3*		//	DUPLICATED
A060					*VPS26B*		//	DUPLICATED
A060					*THYN1*		//	DUPLICATED
A060					*ACAD8*		//	DUPLICATED
A060					*B3GAT1*		//	DUPLICATED
A060		Yp11.32(1421350_1634267)x2 pat	212.9 kb	VOUS	*IL3RA*		//	INT START
A060					*SLC25A6*		//	DUPLICATED
A060					*ASMTL*		//	DUPLICATED
A060					*P2RY8*		//	DUPLICATED
A065	M	9q33.1(119387004_119527204)x1 mat	140.2 kb	LP	*TRIM32*	*TRIM32*	3	DELETED
A065					*ASTN2*	*ASTN2*	2	INTRAGENIC
A065		10q23.1(84137861_84181161)x1 pat	43.3Kb	VOUS	*NRG3*	*NRG3*	//	INTRAGENIC
A066	F	16q24.3(89428111_89453232)x3 pat	25.12 kb	VOUS	*ANKRD11*	*ANKRD11*	1	INTRAGENIC
A078	M	5q11.2(51915987_53470988)x1 mat	1.56 Mb	VOUS	*PELO*		//	DELETED
A078					*ITGA1*		//	DELETED
A078					*ITGA2*		//	DELETED
A078					*MOCS2*		//	DELETED
A078					*FST*		//	DELETED
A078					*NDUFS4*		//	DELETED
A078					*ARL15*		//	DELETED
A078		6q27(165421392_165801341)x3 pat	335.6 kb	VOUS	*PDE10A*	*PDE10A*	//	INT END
A079	M	1p32.1(59947236_60012581)x1 mat	65.35 kb	VOUS	*FGGY*	*FGGY*	//	INTRAGENIC
A080	M	6p21.32(32179815_32312148)x1 mat	132,33 Kb	VOUS	*NOTCH4*	*NOTCH4*	//	DELETED
A080		11q14.1(84455989_84607402)x1 mat	151.4 Kb	LP	*DLG2*	*DLG2*	2	INTRAGENIC
A082	M	15q11.2(25226260_25241828)x3 mat	15.57 kb	VOUS	*SNURF*	*SNURF*	//	INTRAGENIC
A082					*SNHG14*		//	INTRAGENIC
A082		16p13.3(7041435_7132676)x1 pat	91.24 kb	LP	*RBFOX1*	*RBFOX1*	2	INTRAGENIC
A088	M	Xp22.33(1189595_1383266)x2 o Yp11.32(1162459_1322464)x2	192.42	VOUS	*CRLF2*	*CRLF2*	//	DUPLICATED
A093	M	7q36.3(158801633_158903310)x3 mat	101.68 kb	VOUS	*VIPR2*	*VIPR2*	//	INT END
A095	M	Xp22.31(7532513_8115193)x2 mat	582.68 Kb	VOUS	*VCX*		//	DUPLICATED
A095					*PNPLA4*		//	DUPLICATED
A097	M	Xp22.33(169791_537202)x2 mat	367.41 kb	VOUS	*PLCXD1*		//	DUPLICATED
A097					*GTPBP6*		//	DUPLICATED
A097					*PPP2R3B*		//	DUPLICATED
A099	M	3q29(196418238_196487740)x3 pat	69.5kb	VOUS	*PIGX*		//	INT START
A099					*PAK2*	*PAK2*	2	INT END
A107	M	13q14.11(43558077_43731680)x3 mat	173.6 kb	VOUS	*EPSTI1*		//	INT START
A107					*DNAJC15*		//	DUPLICATED
A110	M	2q23.1(148805626_148936476)x1 mat	130.85 kb	LP	*MBD5*	*MBD5*	1	INTRAGENIC
A113	M	13q12.11(20413829_20445336)x3 mat	31.51 Kb	VOUS	*ZMYM5*	*ZMYM5*	//	INT START
A119	M	Yp11.32 (771993_1322464)x2 pat	550 Kb	VOUS	*CRLF2*	*CRLF2*	//	DUPLICATED
A120	M	8q24.3(144825929_144881529)x3 pat	55.6 Kb	VOUS	*SCRIB*	*SCRIB*	//	INT END
A120		14q31.1(79388339_79657573)x1 dn	269.24 Kb	P	*NRXN3*	*NRXN3*	1	INTRAGENIC

A121	F	13q32.2(99177988_99271292)x3 pat	93.31 Kb	VOUS	*STK24*	*STK24*	//	INT START
A121	M	4q25(110390080_110653618)x3 mat	263.54 Kb	VOUS	*SEC24B*	*SEC24B*	//	INT START
A121					*CASP6*		//	DUPLICATED
A121					*PLA2G12A*		//	DUPLICATED
A121		4q25(113167151_113610549)x3 mat	443.4 Kb	VOUS	*AP1AR*		//	INT START
A121					*TIFA*		//	DUPLICATED
A121					*ALPK1*		//	DUPLICATED
A121					*NEUROG2*	*NEUROG2*	//	DUPLICATED
A121					*LARP7*		//	DUPLICATED
A124	M	1q21.1q21.2(145804790_147860611)x3 pat	2.06 Mb	LP	*GPR89A*		//	INT START
A124					*NBPF11*		//	DUPLICATED
A124					*HYDIN2*	*HYDIN2*	2	DUPLICATED
A124					*NBPF12*		//	DUPLICATED
A124					*PRKAB2*		//	DUPLICATED
A124					*FMO5*		//	DUPLICATED
A124					*BCL9*		//	DUPLICATED
A124					*CHD1L*		//	DUPLICATED
A124					*ACP6*		//	DUPLICATED
A124					*GJA5*		//	DUPLICATED
A124					*GJA8*		//	DUPLICATED
A124					*GPR89B*		//	DUPLICATED
A124		12q24.12q24.13(112184121_112302954)x3 pat	118.83 Kb	VOUS	*ACAD10*		//	INT START
A124					*ALDH2*		//	DUPLICATED
A124					*MAPKAPK5*		//	INT END
A125	M	Xq12(65768203_65926180)x2 mat	155.7 Kb	VOUS	*EDA2R*	*EDA2R*	//	DUPLICATED
